# Unraveling the effects of the gut microbiota composition and function on horse endurance physiology

**DOI:** 10.1038/s41598-019-46118-7

**Published:** 2019-07-03

**Authors:** Sandra Plancade, Allison Clark, Catherine Philippe, Jean-Christophe Helbling, Marie-Pierre Moisan, Diane Esquerré, Laurence le Moyec, Céline Robert, Eric Barrey, Núria Mach

**Affiliations:** 1 MaIAGE, INRA, Université Paris-Saclay, Jouy-en-Josas, France; 2 ISBA, Université Catholique de Louvain, Louvain-la-Neuve, Belgium; 3 Gastroenterology Department, Vall d’Hebron Institut de Reserca, Barcelona, Spain; 4 UMR 1319, INRA, AgroParisTech, Université Paris-Saclay, Jouy-en-Josas, France; 5 UMR 1286, INRA, Université Bordeaux, Nutrition et neurobiologie intégrée, Bordeaux, France; 6 UMR 444, INRA, Plateforme GET, Castanet-Tolosan, France; 7 Unité de Biologie Intégrative et Adaptation à l’Exercice, UBIAE, EA7362, Université d’Evry, Université Paris-Saclay, Evry, France; 8 UMR 1313, INRA, AgroParisTech, Université Paris-Saclay, Jouy-en-Josas, France; 9 Ecole Nationale Vétérinaire d’Alfort, Maisons-Alfort, France

**Keywords:** Homeostasis, Metagenomics

## Abstract

An integrated analysis of gut microbiota, blood biochemical and metabolome in 52 endurance horses was performed. Clustering by gut microbiota revealed the existence of two communities mainly driven by diet as host properties showed little effect. Community 1 presented lower richness and diversity, but higher dominance and rarity of species, including some pathobionts. Moreover, its microbiota composition was tightly linked to host blood metabolites related to lipid metabolism and glycolysis at basal time. Despite the lower fiber intake, community type 1 appeared more specialized to produce acetate as a mean of maintaining the energy supply as glucose concentrations fell during the race. On the other hand, community type 2 showed an enrichment of fibrolytic and cellulolytic bacteria as well as anaerobic fungi, coupled to a higher production of propionate and butyrate. The higher butyrate proportion in community 2 was not associated with protective effects on telomere lengths but could have ameliorated mucosal inflammation and oxidative status. The gut microbiota was neither associated with the blood biochemical markers nor metabolome during the endurance race, and did not provide a biomarker for race ranking or risk of failure to finish the race.

## Introduction

Endurance athletes endure physiological and biochemical disruptions that require the body to undergo several adaptations to maintain homeostasis^1–5^, which results in neuromuscular and contractile functions in muscles^6^, electrolyte imbalance^6^, decreased glycogen storage^6^, increased mitochondrial biogenesis in muscle tissue^7^, increased oxidative stress^4^, activation of the sympathetic-adrenomedullary and hypothalamus-pituitary-adrenal (HPA) axes, increased intestinal permeability as well as inflammatory responses (reviewed by Clark and Mach^1^). Consequently, adaptations to endurance exercise are influenced by the transcriptional and translational regulation of genes that encode the proteins and metabolites controlling these processes^4,8^. Recent metagenomic studies have revealed that intense physical exercise also induces changes in the gut microbiome^9–13^.

The equine microbiota reaches a concentration of approximately 10^9^ microorganisms per gram of ingesta in the cecum^14^, and consists of about 108 bacterial genera^15–17^ and at least seven phyla^17–22^. The variation in the gut microbiota composition and functions in endurance equine athletes is not clear. For example, it is unknown whether inter-individual variation results from a continuum of different bacterial compositions or whether individual gut microbiota gather around some stable bacterial communities that can be associated with athletic performance. Studying such questions is complicated due to the complexity of varying physiological (*e*.*g*. breed, age, sex, host kinship), nutritional and environmental conditions between individuals. In fact, diet dramatically modulates gut microbiota composition and metabolism in horses^16,19,23–29^. Endurance horses often consume diets high in fermentable carbohydrates that provide a continuous source of energy during sustained physical exercise^28,30^. However, the over consumption of readily fermentable carbohydrates can reduce microbiota diversity and function (*e*.*g*. less synthesis of byproducts such as short chain fatty acids (SCFA) and secondary bile acids), which may play a role in the development of common equine intestinal diseases^28,30^.

Given the gut microbiota’s fundamental role in maintaining host homeostasis during endurance exercise, we first aimed to study similarities and differences between the gut microbiota among endurance athletes. We then sought to identify the possible relationship between blood biochemical and metabolic profiles and the gut microbiota composition to (i) reveal unique biomarkers of the energy and stress response to endurance exercise, as well as athletic performance and (ii) provide insights into the molecular control of this response. We therefore performed an integrated analysis of the blood biochemical and metabolic profiles in 52 endurance horses (racing over 90, 120 and 160 km) before and after an endurance competition as well as analysis of the gut microbiome before the race while considering diverse physiological and nutritional factors.

## Results

A total of 52 endurance horses (out of 248) racing over 90, 120 or 160 km were recruited in a volunteer base at an endurance competition in Fontainebleau (France; Supplementary Table S1 and Fig. S1). Fecal samples with time-matched blood and questionnaire data (with emphasis on estimated nutrient consumption) were analyzed. Encompassing an equal range of race distance, age, sex, breeds, heart rate, speed data, athletic performance ranking, genetic background and environmental factors, our cohort was expected to be representative of the average gut microbiota composition of endurance horses. Nevertheless, the endurance rules impose a minimum age of 7 years for 120 km and 8 years for 160 km races, therefore excluding young horses (<7 years old) in these two distance groups. For detailed metadata, see Supplementary Table S1 and Supplementary Data.

Only one Arabian female of 10 years (namely “EcaOmic.201”) was excluded from the cohort as it presented very heterogeneous and distinctive gut microbiota and distorted ordination solutions. This single sample was removed from the study after considering various measures of richness, evenness, diversity, dominance, rarity, divergences and abundance (Supplementary Table S2).

### Description of the gut microbiota in endurance equine athletes

#### Gut phylogenic composition and core microbiota in horse

Fecal samples were obtained before the race to profile the gut microbiome using 16S ribosomal RNA gene amplicon sequencing. The overall phylogenic composition of the sequenced samples confirmed that the Firmicutes (60.1 ± 3.70%) outranked Bacteroidetes (19.3 ± 4.28%), Fibrobacteres (9.6 ± 5.11%) and Spirochaetes (8.9 ± 2.26%) phyla. *Lachnospiraceae* (27.4 ± 2.51%), *Ruminococcaceae* (24.78 ± 4.29%), *Fibrobacteraceae* (9.7 ± 5.26%), *Spirochaetaceae* (9.1 ± 2.33%) and *Prevotellaceae* (8.37 ± 2.02%) were the most abundant families across samples. At the genus level, *Clostridium* XIVa (16.2 ± 2.34%), *Fibrobacter* (11.0 ± 5.81%) and *Treponema* (10.3 ± 2.57%) accounted for most of gut microbiota composition. The relative abundance of each genus in our cohort is shown in Supplementary Table S3.

The endurance horse core microbiota (*e*.*g*. the genera shared by 99% of samples with a minimum detection threshold of 0.001%) composed of 23 genera, including *Clostridium XIVa*, *Fibrobacter*, *Treponema*, *Ruminococcus*, and members of the yet unclassified *Lachnospiraceae* family (Supplementary Fig. S2A,B). *Alloprevotella*, *Anaerovibrio*, *Desulfovibrio*, *Paludibacter*, *Streptococcus*, and members of the unclassified *Ruminococcaceae* family were not detected in the core microbiota although they were between the top 30 most abundant genera in our cohort (Supplementary Table S3).

#### Inter-individual variation: the gut microbiota was structured into two-community types

**Figure 1:**
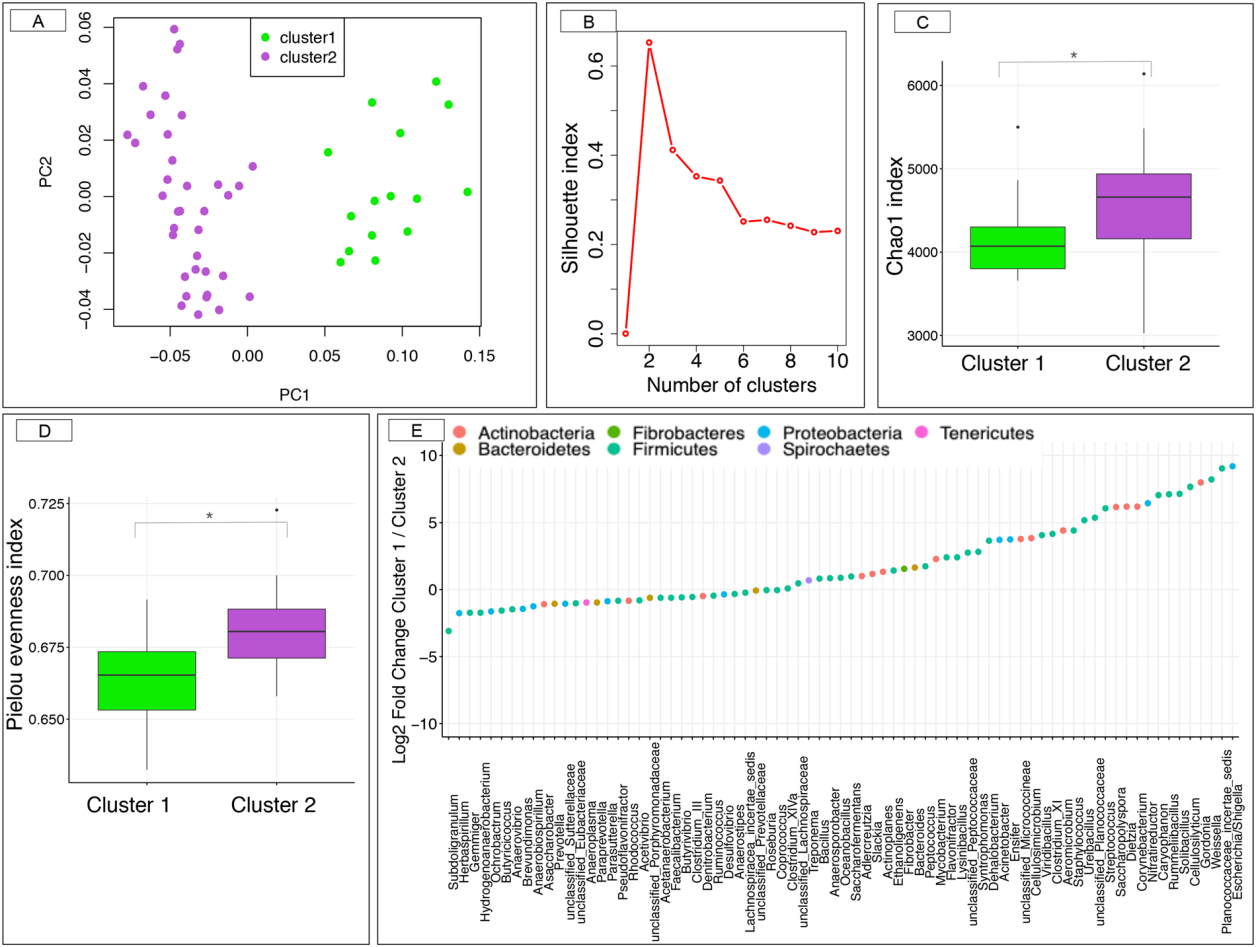
Estimation of the α- and β-diversity within the cohort of study. (**A**) Principal coordinate analysis (PCoA) with weighted UniFrac distance based on the OTUs table. Color corresponds to the gut microbiota community types based on the medoids (PAM) algorithm (green = community type 1, and purple = community type 2). The first community contained 15 individuals whereas the second community included 36 individuals. (**B**) The number and quality of clusters were validated by maximizing the silhouette index; (**C**) Box plot graph representation of the α-diversity indexes (Chao1) using the rarefied OTU table for each community type; (**D**) Box plot graph representation of the Pielou’s evenness index using the rarefied OTU table for each community type. In all cases, boxes show median and interquartile range, and whiskers indicate 5^th^ to 95^th^ percentile. Community type 1 animals are colored in green and community type 2 animals in purple; * adjusted *p*-value < 0.05, Mann Whitney *U* test followed by Benjamini-Hochberg multiple test correction; (**E**) Dot plot representation of log-transformed fold change of genera that were significantly different between the community type 1 and type 2. The logs of fold changes lying between 0 and 10 indicate that genera were more abundant in community type 1 than community type 2. By contrast, the logs of fold changes lying between 0 and −10 indicate that the genera abundances were lower in community type 1 compared to community type 2. Dots are colored by phyla.

The Principal Coordinate Analysis (PCoA) based on weighted UniFrac distance (Fig. 1A) showed a distinct two community structure between samples, numerically confirmed by the partitioning around medoids (PAM) silhouette coefficient using weighted UniFrac distance of the normalized open-reference operational taxonomic (OTUs) counts (Fig. 1B). PAM algorithm with alternative dissimilarity measures produced the exact same communities, assessing the robustness of the community structure of the microbiota. The robustness of the community types was further studied through alternative reduction dimension approaches, which led to identical observations (Supplementary Fig. S3A–D).

#### The cohort characteristics and the phylogenetic and functional bases of the two gut microbiota community types were substantially different

The first community (community type 1), which contained 15 individuals, was predominantly composed of Arabian geldings that raced over 160 km, individuals with and average age of 9.4 ± 2.41 years, average speed for the entirety of the race of 16.7 ± 1.68 km/h and pulse rate on arrival at the veterinary check of 56.14 ± 3.57 beats/min (Supplementary Fig. S4A–F). Four participants were classified as the top 25% athletes, and 33% of them were eliminated from the race (Supplementary Fig. S4G). In contrast, community type 2, consisting of 36 individuals, mainly comprised females and geldings that primarily raced 90 km or 120 km. The type 2 individuals were 9.2 ± 2.08 years old, and their average speed and pulse rate on arrival were 17.2 ± 1.60 km/h and 54.53 ± 5.7 beats/min, respectively (Supplementary Fig. S4A–G). Eleven individuals were classified within the top 25%, and 16% were eliminated from the race. Trainers, stables and breeding establishments were equally represented between community types.

At the OTUs level, the community type 1 presented significantly reduced microbiome richness, diversity and most of the evenness indices but higher dominance and rarity of non core and rare species (adjusted *p-*value < 0.05, Mann-Whitney *U* test) compared with the community type 2 (Supplementary Fig. S5). For example, Chao 1 (Fig. 1C) and Pielou index (Fig. 1D), which is an excellent measure of community structure, were significantly lower in community type 1 relative to community type 2. The significant differences in taxon evenness and rarity were found up to the genera level. By contrast, the Chao1 index based on the genera level was higher (*p-*value = 3.10^−6^, Mann-Whitney *U* test) in the community type 1 relative to the community type 2.

**Figure 2:**
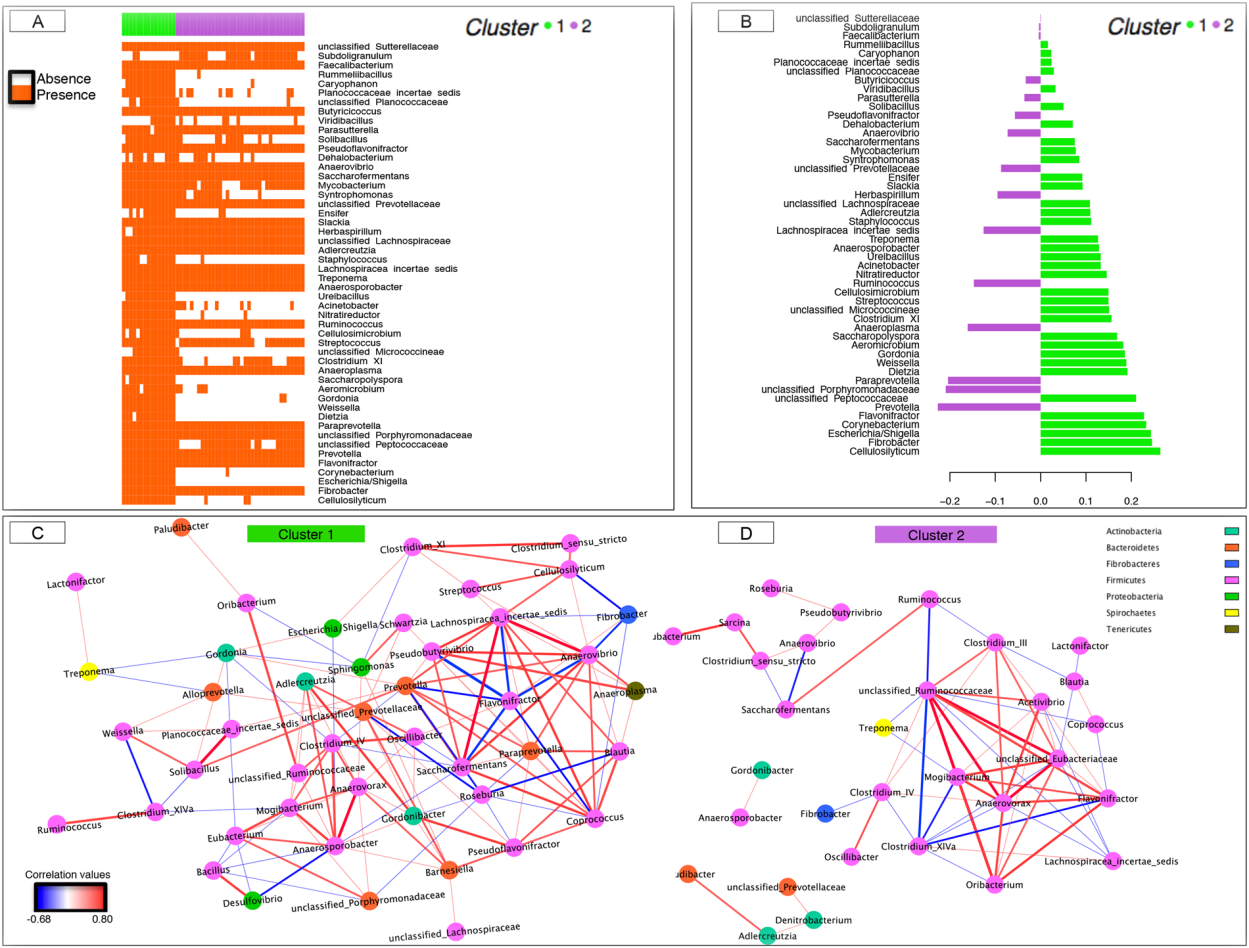
Dynamics of gut bacterial genera between the two community types. (**A**) Matrix showing the presence or absence of the 50 most discriminating genera detected by partial least squares discriminant analysis (PLS-DA) model. Each entry in the matrix indicates the presence or absence of each genus in each individual. Individuals are grouped by community type. In the heatmap, orange = presence, white = absence; (**B**) The PLS-DA loading plot shows the contributing bacterial genera towards the separation of the PLS-DA scores between individuals of the community type 1 (green color) and community type 2 (purple color); (**C**,**D**) Co-occurrence network of the community type 1 and type 2, respectively. In all cases, the correlations among genera were calculated using the partial correlation and information theory (PCIT) method, which identifies significant co-occurrence patterns. The size of the node is proportional to genera abundance. Node fill color corresponds to phylum taxonomic classification. Edges colors represent positive (red) and negative (blue) connections. The edge thickness is equivalent to the correlation values. Only genera with a relative abundance >0.10 were included.

Following the findings of evident differences in the ecosystem diversity, we further investigated the composition and functionality of the gut microbiota through the fecal SCFA and pH. The relative abundance of 76 genera differed significantly between gut microbiota community types (adjusted *p-*values < 0.05 with Fisher exact test and Mann-Whitney *U* test combined; Supplementary Table S4). Community type 1 was characterized by a lower proportion of Bacteroidetes and Proteobacteria-associated significant taxa but higher proportions of Actinobacteria and Firmicutes phyla (Fig. 1E). Type 1 individual’s harbored a lower proportion of cellulolytic bacteria (members of the *Ruminococcaceae* and *Lachnospiraceae* families) proteolytic bacteria (*e*.*g*. *Prevotella*) and butyrate producer (*e*.*g*. *Butyrivibrio*), but higher proliferation of amylolytic bacteria such as *Streptococcus* (Supplementary Table S4). Moreover, genera such as *Dietzia*, *Escherichia/Shigella*, *Saccharopolypsora*, *Ureibacillus* and *Weissella* appeared exclusively in the community type 1 (Fig. 2A), whereas other genera such as *Actinoplanes*, *Bacteroides*, *Caryophanon*, *Corynebacterium*, *Rummeliibacillus*, *Staphylococcus* and members of the *Micrococcineae* family were present in less than 3% of the individuals from the community type 2 (Fig. 2A). These aforementioned genera were also detected as discriminant between community types when using the partial least squares discriminant analysis (PLS-DA; Fig. 2B). Supporting the later results, the adjusted *p-*values of the Fisher exact test and the Mann-Whitney *U* test combined appreciably decreased with the increased loading values in the PLS-DA model (Supplementary Fig. S6A).

The sparse PLS-DA in a cross-validation framework suggested that 7 genera were sufficient to predict the two gut communities, namely *Cellulosilyticum*, *Corynebacterium*, *Escherichia/Shigella*, *Fibrobacter*, *Flavonifractor*, *Prevotella* and members of the family *Peptococcaceae* (Supplementary Fig. S6B), while the sparse *k-*means suggested that *Cellulosilyticum*, *Corynebacterium* and *Escherichia/Shigella* were enough to almost recover the two gut microbiota communities. Nevertheless, when applying an alternative clustering classification method, which removed the *k* genera with the highest absolute loading values in the PLS-DA, we observed that the exact two community types were maintained even if the 19 genera with the highest loading values were removed (Supplementary Fig. S6C). The PCoA performed on relative genera abundances after deleting the top-rank genera (from *k* = 10 to *k* = 50) was able to discriminate the same two community types (Supplementary Fig. S6D).

The phylogenetic differences among the two community types were reflected on the co-occurrence networks (based on partial correlation and information theory, PCIT). The co-occurrence of the community type 1 was more complex and dense (139 associations among 46 genera; Fig. 2C) than that of the community type 2 (64 associations among 31 genera; Fig. 2D). While both networks roughly followed a scale-free degree distribution (0.94 and 0.70 for community type 1 and 2, respectively), the betweenness centrality scores for community type 1 was higher relative to community type 2 (Supplementary Table S5), as well as other node-level topological measures for each node, including degree and cluster coefficient. Such distinct topological features were additionally used to highlight key genera in co-occurrence networks. The genera showing the largest degree and betweenness centrality values in the community type 1 were *Prevotella* outranked by members of the family *Lachnospiraceae*, *Anaerosporobacter*, *Anaerovibrio*, *Gordonia* and *Coprococcus* (Fig. 2C). On the other hand, a large fraction of the putative key genera in the community type 2 were related to the phylum Firmicutes, including members of the family *Ruminococcaceae*, *Eubacteriaceae* and *Lachnospiraceae*, as well as *Anaerovorax* and *Clostridium* XIVa (Fig. 2D).

**Figure 3:**
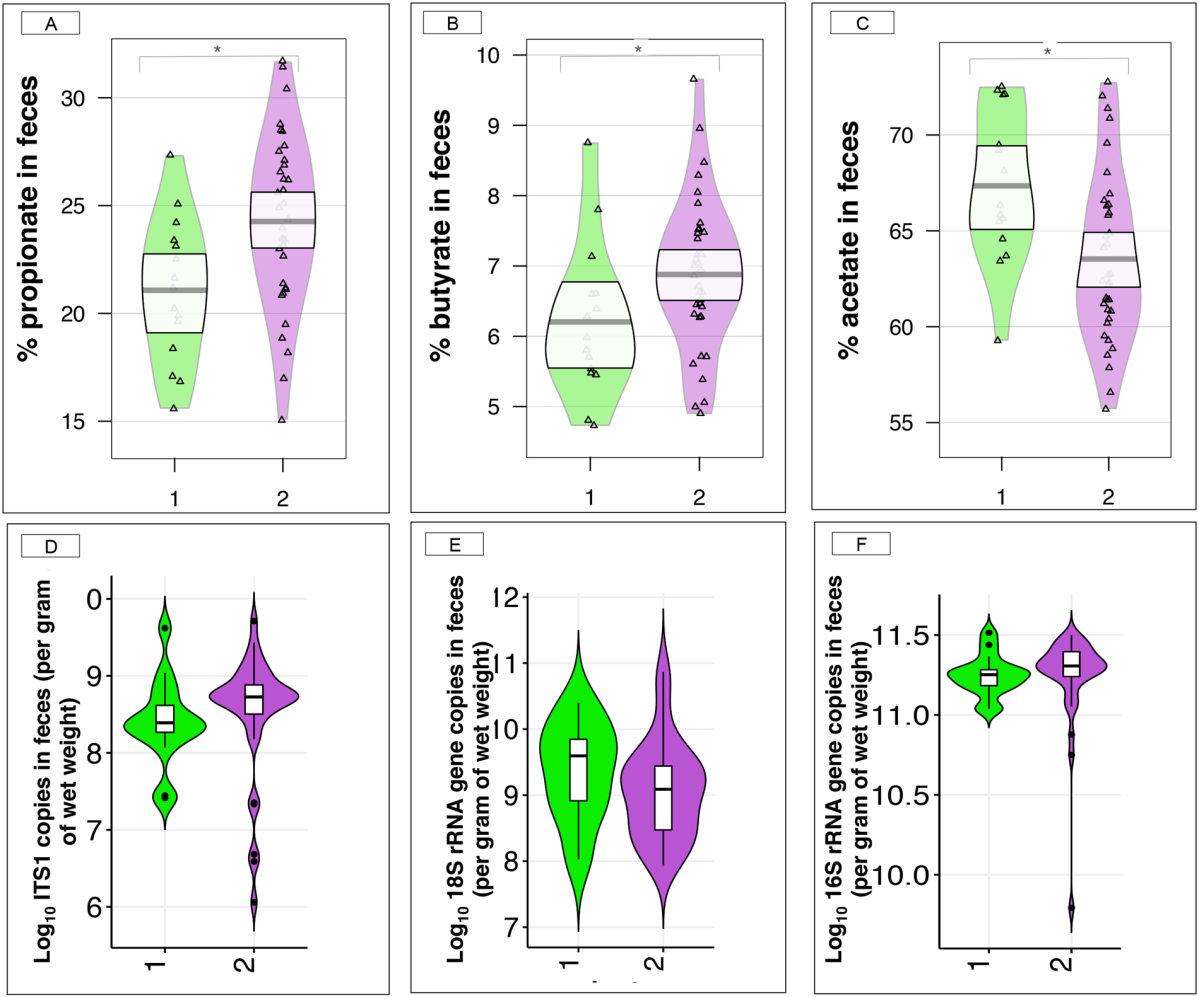
Proportions of short chain fatty acids, and anaerobic fungal, protozoan and bacterial loads in feces of the two-gut microbiota community types. (**A**–**C**) Box plot of the proportion of short chain fatty acids that were significantly different between the community types 1 and community type 2 (Mann Whitney *U* test, adjusted *p*-value < 0.05), namely propionate, butyrate and acetate; (**D**–**F**) Box plot of anaerobic fungal, protozoan and bacterial loads in community type 1 and community type 2, respectively. In all cases, boxes show median and interquartile range, and whiskers indicate 5^th^ to 95^th^ percentile. The box color indicates the community type: community type 1 (green) and community type 2 (purple). * adjusted *p*-value < 0.05, Mann Whitney *U* test followed by Benjamini-Hochberg multiple test correction.

When focusing on the microbiota functionality, we observed that propionate (adjusted *p*-value = 0.02, Mann-Whitney *U* test, Fig. 3A) and butyrate (adjusted *p*-value = 0.05, Mann-Whitney *U* test, Fig. 3B) proportions in feces were higher in individuals from community type 2, while acetate proportion was higher (adjusted *p*-value = 0.024; Mann-Whitney *U* test, Fig. 3C) in the community type 1 relative to community type 2. *Iso*-butyrate, valerate and *iso*-valerate were at similar concentrations between communities, as well as the feces pH. More information on gut physiology parameters variations can be found in Supplementary Table S6.

The study of concomitant microbial populations in the gut showed that the anaerobic fungal loads in feces tended (*p*-value = 0.07, Mann-Whitney *U* test) to be greater in community type 2 (Fig. 3D), while the protozoan (Fig. 3E) and bacteria (Fig. 3F) loads remained unchanged (*p*-value = 0.14, Mann-Whitney *U* test) between gut community types.

### The relationship between gut microbiota, host variables and environmental factors

#### Gut community types were not associated with host variables but rather estimated nutrient intake and composition

**Figure 4:**
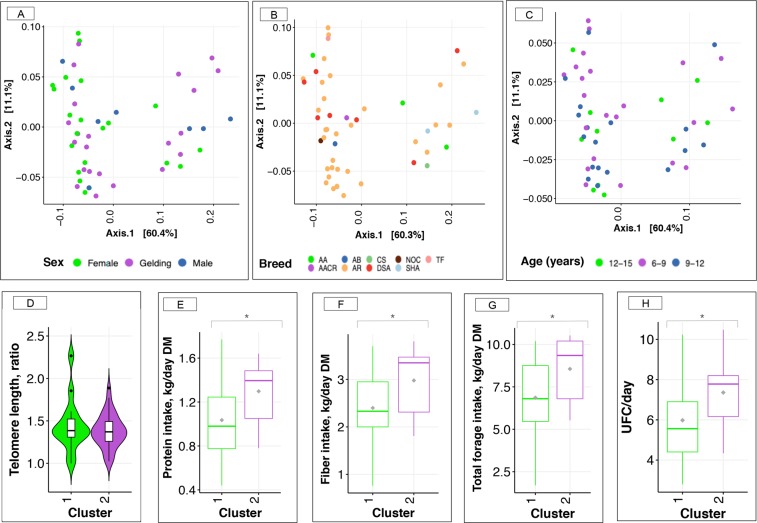
Relationship between microbiota gut community types and host variables. (**A**–**C**) Principal coordinate analysis (PCoA) of gut microbiota composition with weighted-UniFrac distance as a function of sex, breed and category of ages. Within breed: AA (Anglo-Arabian), AB (barbe Arabic), CS (riding horse, half Arabian), NOC (Half Arabian, half unknown), TF (French trotter), AACR (cross breeding Anglo -Arabian), AR (Arabian), DSA (50% Arabian blood), and SHA (Shagya); (**D**) Box plot of telomere length in gut community types 1 and 2; (**E**–**H**) Box plot of estimated daily protein intake (kg), fiber intake (kg), the total forage intake (kg) and UFC intake in gut community types 1 and 2, respectively. The protein, fiber and forage intakes are expressed in dry matter bases. In all cases, boxes show median and interquartile range, and whiskers indicate 5^th^ to 95^th^ percentile. The box color indicates the community type: community type 1 (green) and community type 2 (purple).

Horse sex (*p*-value = 0.36), breed (*p*-value = 0.17) and age of the athletes (*p*-value = 0.91) were neither associated with gut community types (Fisher exact test for categorical variables and Mann-Whitney *U* test for continuous variables) nor with the relative genera abundance matrix (Fig. 4A–C). *Post hoc* power analysis showed that for the variable sex and breed, a sample size of 330 and 100 individuals, respectively, should be necessary to achieve a type II error of 0.1 for the observed effect size in our cohort, whereas a sample size of more than 1,000 individuals should be required to reveal a significant association between age and gut microbiota community types with a type II error of 0.1, given the empirical effect size. For the age variable, the sample size of our cohort would allow detecting an effect size 2 times larger than the observed effect for a type II error of 0.1.

The telomere lengths (*p*-value = 0.58, Fig. 4D) and the host kinship (*p*-value = 0.67) were unchanged between the two gut community types (Mann-Whitney *U* test).

Unlike host variables, the subsequent permutational multivariate analysis of variance (PERMANOVA) demonstrated that the factor most strongly associated with community variation was the estimated diet composition (*e*.*g*. protein, fiber, fat and ash intake corrected by the horse net energy value of feeds (UFC); *p*-value = 0.04, PERMANOVA). At the level of single nutrients, estimated protein (adjusted *p*-value = 0.05, Fig. 4E), fiber (adjusted *p*-value = 0.05, Fig. 4F) and ash (adjusted *p*-value = 0.05) daily intakes were significantly higher in community type 2 relative to community type 1 (Mann-Whitney *U* test, Supplementary Table S6). They were all significant probably because individuals with the community type 2 had a greater forage intake (adjusted *p*-value = 0.05, Mann-Whitney *U* test, Fig. 4G) compared to individuals from community type 1, which consequently accounted for a large portion of UFCs (adjusted *p*-value = 0.05, Mann-Whitney *U* test, Fig. 4H) in their diet. Because diet composition and intakes might be impacted by the fact that training intensity, duration and frequency were different between endurance horses racing over 90, 120 or 160 km, we assessed whether the differences observed between community types and nutrient and energy intakes were subject to be influenced by the race distance. None of the nutrients or the UFC intakes was significantly associated with race distance covered when corrected by the gut microbiota community type effect (*p*-value = 0.6, two-way ANOVA).

Other environmental factors such as hours traveled to arrive at the competition site (*p-*value = 0.49, Mann-Whitney *U* test), the stable from where they were coming (*p-*value = 0.74, Fisher exact test), trainer (*p-*value = 0.74, Fisher exact test) and the breeding establishment where they were born and kept together with their mothers until they were weaned (*p-*value = 0.13, Fisher exact test) were not significantly related to gut microbiota community types.

#### The gut microbiota was related to the biochemical and metabolomic profiles before the endurance race but had no role in the endurance performance

The basal profiles of total bilirubin, creatine kinase, aspartate transaminase, serum amyloid A, as well as non-esterified fatty acids and β-hydroxy-butyrate were not affected by the microbiota gut community types (*p*-value = 0.85; PERMANOVA test, Supplementary Table S7). Similarly, these biochemical variables were not associated with the genus-level community ordination (non-metric multidimensional scaling (NMDS) based on Bray-Curtis dissimilarity) when using the *envfit* function in the “vegan” R package (10,000 permutations, Benjamini-Hochberg multiple test correction <0.05).

**Figure 5:**
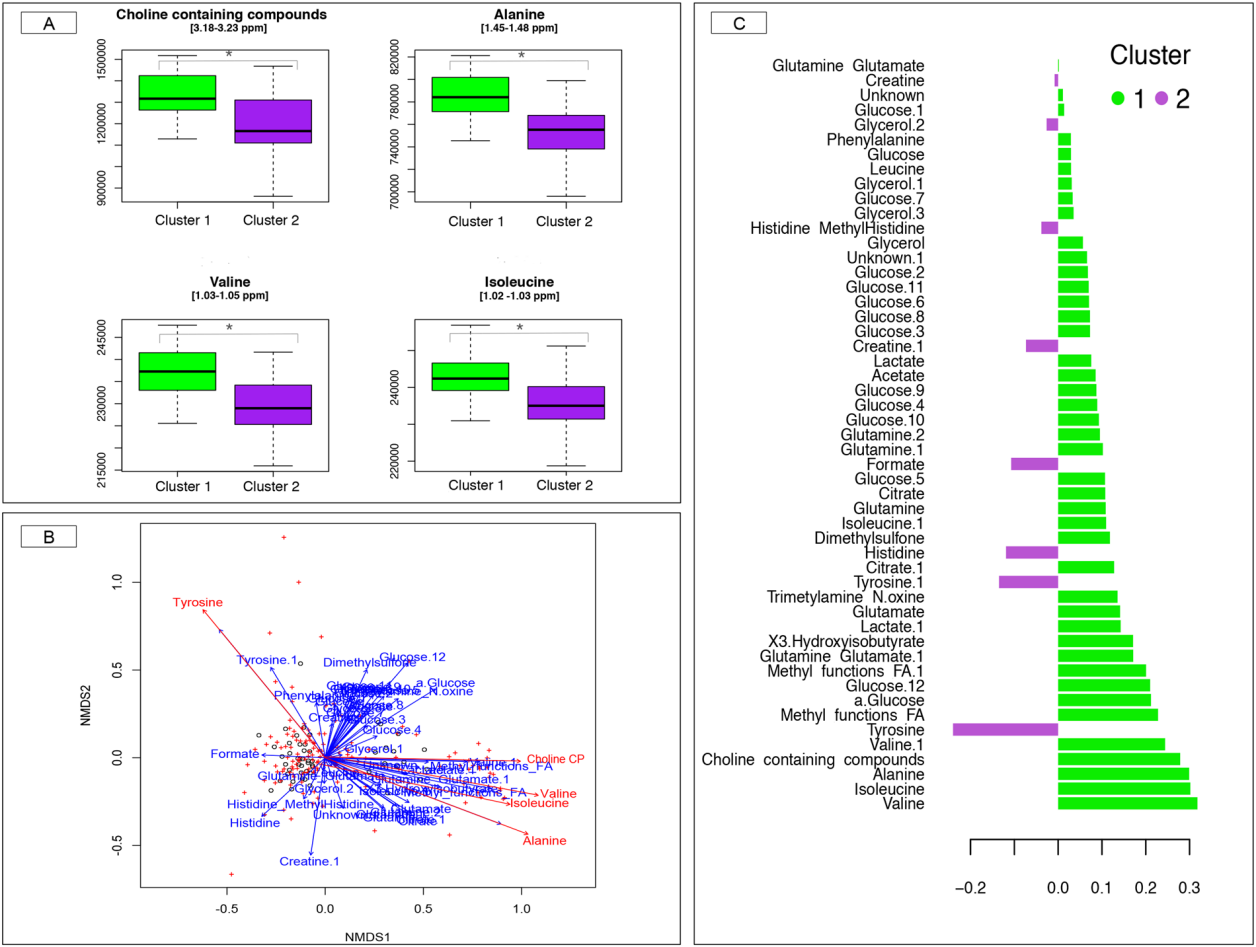
Relationship between blood metabolome profile and gut microbiota at basal time. (**A**) Box plots of blood metabolites significantly different between the two communities’ types at basal time. Boxes show median and interquartile range, and whiskers indicate 5^th^ to 95^th^ percentile. The box color indicates the community type: community type 1 (green) and community type 2 (purple). * adjusted *p-*value < 0.10, Mann Whitney *U* test followed by Benjamini-Hochberg multiple test correction; (**B**) Covariates of microbiome variation were identified by calculation the association between basal metabolome profiles and genus-level community ordination (NMDS based on Bray-Curtis dissimilarity) with *envfit* function in the “vegan” R package (10,000 permutations, followed by Benjamini-Hochberg multiple test correction). Points represent the basal metabolites parameters, whereas crosses represent the relative genera abundance. The blue arrows indicate non-significant correlations with the ordination, whereas the red arrows indicate significant correlations; (**C**) The partial least squares discriminant analysis (PLS-DA) loading plot shows the contributing blood metabolites towards the separation of the PLS-DA scores between individuals of the community type 1 (green color) and community type 2 (purple color) at basal time.

Conversely, the basal metabolome profile (determined using the untargeted broad-window nuclear magnetic resonance; ^1^H NMR) was significantly associated (*p*-value = 0.024, PERMANOVA test) with the gut community types. At the level of single metabolites, we observed that blood alanine (adjusted *p*-value = 0.03) and valine (adjusted *p*-value = 0.05) significantly co-varied with gut community types, while the adjusted *p-*value for choline containing compounds (adjusted *p*-value = 0.07) and isoleucine (adjusted *p*-value = 0.06) were very close to the defined threshold of 0.05 (Mann-Whitney *U* test). All these metabolites were higher in the community type 1 relative to the community type 2 (Fig. 5A). Similarly, the blood basal levels choline containing compounds, alanine, valine, tyrosine and isoleucine were associated with the genus-level community ordination (NMDS based on Bray-Curtis dissimilarity) when using the *envfit* function (10,000 permutations, BH multiple test correction <0.05; Fig. 5B). The aforementioned associations were also confirmed by PLS-DA (Fig. 5C). Further information on blood metabolite profiles is depicted in Supplementary Data, Fig. S7 and Table S8.

Given that basal metabolome profiles are likely related to racing experience, racing training intensity and duration, we assessed whether those differences observed between gut community types on the basal molecules represented an indirect means by which race distance conditioned gut microbiota composition. None of the basal variables were significantly associated with distance, except for the choline containing compounds concentration (*p-*value = 0.04, two way ANOVA; Supplementary Table S9).

Unexpectedly, the biochemical (*p*-value = 0.90, PERMANOVA test) and metabolic (*p*-value = 0.35, PERMANOVA test) profiles during the endurance race were not associated with the gut microbiota communities, not with the genus-level community ordinations.

Beyond the lack of the biochemical and metabolomic regulations of the gut microbiota during the endurance race, we also observed that the microbiota community types were neither linked to the risk of being eliminated during the competition (*p*-value = 0.26, Fisher exact test; Supplementary Fig. S8A) nor the endurance race ranking (*p*-value = 0.62, Fisher exact test, Supplementary Fig. S8B). A sample size of 340 (for elimination) to 700 (for race ranking) would be necessary to achieve a type II error of 0.1 with the observed size effect and a probability of 0.9 (Supplementary Fig. S9A-B). Moreover, the sample size of our cohort would allow detecting an effect size 2 to 3 times larger than the observed effect for a type II error of 0.1 (Supplementary Fig. S9C-D). Detailed calculations on statistical power and effect size are reported in Supplementary Data.

## Discussion

Results showed that the constituent phyla, families, and genera within the gut microbiota were congruent with other studies performed in horses^15–21,31–34^. *Clostridium* XIVa, *Fibrobacter* and *Treponema* genera were the most abundant genera, which is in agreement with a study in Standardbred racehorses by Janabi *et al*.^35^ that showed that training led to an increase in *Treponema* and *Clostridium* spp.

Microbiome analyses showed that the endurance horses’ gut microbiota composition clustered into two functionally distinct groups, revealing possible diverse metabolic adaptations to endurance exercise between the two gut communities. Community type 1 showed lower richness and diversity at the OTUs level, but appeared more specialized to produce acetate as a mean of maintaining the energy supply as glucose concentrations fell during the race. The lower diversity and richness have been associated with increased energy harvest from food in obese humans^36^. Therefore, it is likely that community type 1 benefits the host during the endurance race proving significant amounts of acetate, the main substrate from fiber fermentation in horses (reviewed by Jansson and Lindberg, 2012^37^).

Conversely, type 2 community presented higher richness and diversity at the OTUs level, anaerobic fungal loads, as well as elevated levels of metabolites that provided an alternate energy source (*e*.*g*. propionate and butyrate). Anaerobic fungi, which degrade fiber thanks to their rhizoids that have the ability to physically penetrate plant structural barriers^23^, could have led to higher concentrations of propionate, the main precursor for gluconeogenesis in animals, as well as butyrate. The higher N-butyrate concentration could have ameliorated gut mucosal inflammation and oxidative status^38^, common occurrences among athletes as body temperature increases and blood pools away from the gastrointestinal tract to periphery muscles and organs such as the heart and lungs during intense physical activity (reviewed by Clark and Mach^1^). Nevertheless, N-butyrate was not associated with protective effects on telomere length.

After discovering that the gut microbiota composition and function were clearly divided between the two communities, we investigated the major drivers influencing gut microbiota communities. The measured host properties (*e*.*g*. age, sex, breed, host kinship and telomere length) and athletic performance parameters (*e*.*g*. pulse rate, average speed during the race, distance covered, ranking and elimination) were not significantly associated with the gut community types. These results may constitute false-negative associations (type II error) as our current study lacks power to properly explain these associations due to the limited number of participants. A greater sample size is required to achieve statistical significance with host and endurance performance outcome measures.

However, a strong correlation occurred between estimated forage and daily energy intake and gut community types. These findings should be interpreted with caution, as nutrient intake estimation could be inaccurate due to owners or riders not remembering the consumption of specific ingredients or incorrectly estimating portion sizes, and the fact that nutrient composition was estimated from INRA food composition tables. The estimated lower forage intakes in community type 1 was in line with the lower relative abundance of important fibrolytic bacteria, including members of the family *Ruminococcaceae*, *Eubacteriaceae* and *Lachnospiraceae*^39^ (identified as hub-based key taxa in the co-occurrence network in type 2 individuals). However, other fibrolytic bacteria like *Fibrobacter* along with the *Treponema*, a hydrogen utilizing microorganism that work with fibrolytic bacteria^23^, were much higher in community type 1 compared to type 2 and were in line with results from other studies^40^, suggesting that the community type 1 harbored less complex but more specialized microbiota to produce acetate as a mean of maintaining the energy supply as the glucose concentrations fell during the race. Pan and colleagues^41^ showed that acetic acid enhanced endurance performance in exercise-trained mice by inducing enzymes involved in fatty acid oxidation.

At basal time, metabolites such as choline containing compounds, alanine and branched chain amino acids positively correlated with the community type 1’s microbiota composition. The link between community type 1 and choline containing phospholipids levels in blood, the most abundant lipids within the cell membrane, further supported that type 1 community metabolic capacity could enhance host energy metabolism during the endurance race. The modulation of the host energy metabolism was also reflected by the relationship between community type 1 and alanine, whose metabolism is involved in the glycolysis pathway. Community type 1 relationship with the branched chain amino acids, which can be converted in a complex mixture of metabolic end products, including ammonia, and SCFA, reinforces its role in regulating the energy balance during the endurance race. The elevated levels of branched chain amino acids (leucine, isoleucine, valine) may have also reduced endurance exercise- induced muscle damage and fatigue^42^.

Despite the complex relationship between the gut microbiota and energy production during endurance exercise, we found no significant association between blood metabolites (especially those associated with mitochondrial oxidative phosphorylation, fatty acid β-oxidation and gluconeogenesis) and gut microbiota composition during the endurance race.

The lack of association between blood metabolites during the race and the gut microbiota was probably due to some possible limits of the 16S rRNA gene sequencing for microbiota profiling or the ^1^H NMR-based approach for metabolomics. On one hand, the relatively short read length of Illumina-generated sequences along with the specific combination of primer pairs could substantially affect the proportion of single merged sequences as well as the accuracy and sensitivity of taxonomic annotation against the reference databases^43^. The resolution of taxonomical classification of sequences based on a limited segment of the 16S rRNA gene, such as the V3-V4 region, is relatively low^44^. One of the approaches to increase the resolution of taxonomical classification would be the use of whole metagenome shotgun sequencing, which assure non-amplification bias and unbiased estimation of taxonomic profiles^44^. On the other hand, the ^1^H NMR approach only detects those metabolites with high concentrations (*e*.*g*. ketone bodies, amino acids, glucose, pyruvate, choline, etc.). Additionally, because most of the metabolites had multiple peaks in the ^1^H NMR spectra, many of which overlapped each other, the estimation of the measure proportional to the concentration of compound could be ambiguous.

Research about the potential role the gut microbiota plays in athletic performance is in its infancy. Further investigation is needed to better understand the role diet plays in modulating the gut microbiota in endurance athletes, and consequently metabolic pathways that attenuate exercise-induced inflammation, oxidative stress, intestinal permeability and energy homeostasis.

## Methods

### Animals

Fifty-two horses (8 males, 22 mares and 22 geldings; mean ± SEM age: 9 ± 1.75 years) were recruited on a volunteer basis from a cohort of 248 participants. We obtained informed consent from horse owners or riders. For successful participation in the study, we required the following criteria: (i) written informed consent; (ii) blood sample collection before and after the race; (iii) feces collection before the race; (iv) absence of gastrointestinal disorders during the four months prior to enrollment; (v) absence of antibiotic treatment during the four months prior to enrollment and absence of anthelmintic medication within 60 days before the race, and (vi) a complete questionnaire about diet composition and intake. Prior to completing the questionnaires, owners and riders were provided with a written description of the procedures. The number of volunteers enrolled in the study was limited because a high percentage of potential volunteers declined to participate due to they worried about the mechanical phlebitis or chemical phlebitis after blood sampling (specially before the endurance race).

We categorized participants based on the race distance: 90 km, 120 km and 160 km (Supplementary Fig. S1). Out of the 91 horses ascribed to the 90 km race, 19 horses met all inclusion criteria. For the 120 km category, we enrolled only 17 horses out of 101, whereas a total of 16 out of 56 horses that raced over 160 km met all inclusion criteria.

For each horse included in this study, we detailed the age, sex, breed, distance covered during the race, whether the horse completed the race and reason for elimination if any (metabolic, lameness, tiredness or other), pulse rate on arrival at the veterinary check point, the average speed during the race, and the race ranking over all animals racing the same distance. The athletic performance ranking of the horse was calculated by taking into account the average speed, recovery time, riding time and the horse and rider’s ability to finish the race. We also recorded environmental factors such as the hours traveled to arrive at the competition site, the stable from where they were coming, the trainer and the breeding establishment where they were born and kept together with their mothers until they were weaned. Lastly, the estimated macronutrient intakes per horse were also detailed (see the section *Nutrient intake estimation* for further details).

Since it has been previously established that gut microbiota profiles might be shaped by host genetics^45^, we used the “kinship2” R package to create the numerator relationship matrix, which estimates the genetic parameters and predicts breeding values between animals. Both pedigree tree and correlation structure matrix are depicted in Supplementary Figs S10 and S11, respectively.

The weather conditions, terrain difficulty and altitude were the same for all participants enrolled in the study as all races (90, 120 and 160 km) took place during the second weekend of October 2015 in Fontainebleau (France). The average air temperature was 15 °C, with a maximum of 20 °C and a minimum of 11 °C. The average air humidity was 88%. No rain was recorded.

All individuals passed the International Equestrian Federation (FEI)’s compulsory examination before the start. During the endurance competition, all animals underwent veterinary checks every 30- to 40-km during the race, followed by recovery periods of 40 to 50 minutes, which is in accordance with the FEI rules on endurance riding. The veterinarians ensured that horses were not suffering from lameness, metabolic troubles or dehydration, and that their heart rate was low enough to start racing another 30 to 40 km. Horses that failed to complete the race were considered as non-finishers or eliminated.

The local animal care and use committee reviewed and approved the study protocol (ComEth EnvA-Upec-ANSES; reference: 11-0041, dated July 12^th^ 2011). All the protocols were conducted in accordance with EEC regulation (n^o^ 2010/63/UE) governing the care and use of laboratory animals, which has been effective in France since the 1^st^ of January 2013. In all cases, the owners and riders provided their informed consent prior to the start of study procedures with the animals.

### Blood sampling and measurements

#### Blood sampling

Blood samples were collected the day before the event (Basal, T0) and immediately after the end of the competition (T1). Whole blood samples before and after the race were taken in EDTA and lithium heparin tubes (BD Vacutainer®, 10 mL) for biochemical assays and in fluoride-oxalate tubes for metabolomics analysis. Serum and plasma samples were maintained at +4 °C until analysis. Plasma samples collected for ^1^H NMR analysis were frozen immediately after centrifugation and stored at −80 °C.

Additionally, at basal time, blood samples for telomere length profiling were obtained from each animal using EDTA tubes (BD Vacutainer®). After centrifugation, the packed blood cells were maintained at −80 °C until DNA extraction.

#### Blood biochemical assays

Sera were assayed for total bilirubin, conjugated bilirubin, total protein, creatinine, creatine phosphokinase, aspartate amino transferase, gamma glutamyl transferase, non-esterified fatty acids, beta hydroxy-butyrate and serum amyloid A levels on a RX Imola analyzer (Randox, UK).

#### Telomere length measurement

The telomere length measurement assay was adapted from the method described by Cawthon and colleagues^46^. For each DNA sample, the ratio between telomere repeat copy number and the single copy interferon **γ** (*IFGM*) was calculated. The ratio was proportional to the average telomere length. The forward primer for the telomere PCR was Tel1F [5′-CGGTTTGTTT GGGTTTGGGTTTGGGTTTGGGTTTGGGTT-3′] and the reverse primer was TelR [5′-GGCTTGCCTTACCCTTACCCTTACCCTTACCCTTACCCT-3′]. Values reported correspond to ∆∆Ct between Ct value of telomeric region (T) amplification and *IFGM* (S) amplification for each sample relative to a random control sample, here the value of the animal “EcaOmic.213”. Further details on the protocol are reported elsewhere^17^.

#### ^1^H Nuclear magnetic resonance (NMR) data acquisition

The plasmas were thawed at room temperature. In the 5 mm NMR tubes, 600 µL of plasma was added with 100 µL deuterium oxide for field locking. The ^1^H NMR spectra were acquired with a Bruker® Advance III spectrometer (Bruker BioSpin, Wissembourg, France) and a 5 mm reversed QXI Z-gradient high-resolution Bruker probe. Proton spectra were acquired at 500 MHz. The water signal was suppressed with a pre-saturation pulse (3.42 × 10^−5^ W) during relaxation delay (3 s) at the water resonance frequency. The signal processing was performed in an automatic routine with an in-housing code using NMRpipe. The processing method included an integral calculation of 0.001 ppm regions from 9.5 to 0 ppm. Each region was referred as bins. The region between 4.5 and 5 ppm corresponding to residual water signal was withdrawn. The ^1^H NMR data was normalized according to the spectra using the probabilistic quotient method^47^ and the bins, corresponding to variables in the statistical analysis, were scaled to unit variance. Further explanation on sample preparation, data acquisition, data quality control, spectroscopic data-pre-processing, and data pre-processing including bin alignment, scaling and normalization are broadly explained elsewhere^48^.

The metabolite identification was then performed by using structure message of metabolites acquired from other available biochemical databases, such as human metabolome database (HMD), http://www.hmdb.ca; KEGG, http://www.genome.jp/kegg/; METLIN, http://metlin.scripps.edu/; Chemical Entities of Biological Interest (http://www.ebi.ac.uk/Databases/); and Lipidmaps (http://www.lipidmaps.org/) and literature^4,48^. Metabolite assignment of each peak was considered when chemical shifts of peaks in the samples were the same as in the publicly available reference databases or literature (with a shift tolerance level of ± 0.005 ppm), in order to counter-act the effects of measurements and pre-processing variability introduced by factors such as pH values and solvents. A manual curation for identified compounds was done by an expert in horse metabolomics^4,48^. Afterwards, the relative abundance of each metabolite was calculated as the area of the peak^49^.

### Feces sampling and measurements

#### Feces sampling

Fresh fecal samples were obtained from all horses while monitoring them before the race (no more than 24 h before starting the race, in all cases). One fecal sample from each animal was collected off the ground immediately after defecation as described by Mach *et al*.^17^. Since most of the horses experienced dehydration after the race, the gastrointestinal emptying was significantly delayed and consequently we were not able to recover the feces after the race.

#### Feces pH and short chain fatty acids (SCFA) concentration

The feces pH was immediately determined after 10% fecal suspension (wt/vol) in saline solution (0.15 M NaCl solution). SCFAs were measured in fecal samples as previously described in Mach *et al*.^17^.

#### Microorganisms DNA extraction

Total DNA was extracted from aliquots of frozen fecal samples (200 mg; 52 samples at rest), using E.Z.N.A.® Stool DNA Kit (Omega Bio-Tek, Norcross, Georgia, USA). The DNA extraction protocol was carried out according to the manufacturer’s instructions (Omega- Bio-Tek, Norcross, Georgia, USA). DNA was then quantified using the Qubit dsDNA HS assay kit (Thermo Fisher Scientific®; Waltham, MA USA).

#### V3–V4 16S rRNA gene amplification

The V3-V4 hyper-variable regions of the 16S rRNA gene were amplified as previously reported in with two rounds of PCR as previously described in Mach *et al*.^17^ and Clark *et al*.^40^. The concentration of the purified amplicons was measured using Nanodrop 8000 spectrophotometer (Thermo Scientific) and the quality of a set of amplicons was checked using DNA 7500 chips onto a Bioanalyzer 2100 (Agilent Technologies, Santa Clara, CA, USA). All libraries were pooled at equimolar concentration in order to generate equivalent number of raw reads with each library. The final pool had a diluted concentration of 5 nM to 20 nM and was used for sequencing. Amplicon libraries were mixed with 15% PhiX control according to the Illumina’s protocol. For this study, one-sequencing run was performed using MiSeq. 500 cycle reagent kit v2 (2 × 250 output; Illumina, USA).

#### V3–V4 16S rRNA gene sequencing and data preprocessing

Sequences were processed using the version 1.9.0 of the Quantitative Insights Into Microbial Ecology (QIIME) pipeline^50,51^ and by choosing the open-reference OTU calling approach^51^.

First, forward and reverse paired-end sequence reads were collapsed into a single continuous sequence according to the ‘fastq-join’ option of the ‘join_paired_ends.py’ command in QIIME. The fastq-join function allowed a maximum difference within overlap region of 8%, a minimum overlap setting of 6 bp and a maximum overlap setting of 60 bp. The reads that did not overlap (~20% of the total) were removed from the analysis. Anomalously joined reads–reads that were too short or too long were excluded according to the expected size of each targeting region (438–469 bp for region V3–V4). The retained sequences were then quality filtered. De-multiplexing, primer removal and quality filtering processes were performed using the ‘split_libraries’_fastq.py command in QIIME. We applied a default base call Phred threshold of 20, allowing maximum three low-quality base calls before truncating a read, including only reads with >75% consecutive high-quality base calls, and excluding reads with ambiguous (N) base calls^52^.

Subsequently, the sequences were clustered into OTUs against the GreenGenes database (release 2013-08: gg_13_8_otus)^53^ by using the uclust^54^ method at a 97% similarity cutoff. The filtering of chimeric OTUs was performed by using Usearch version 6.1^55^ against the GreenGenes reference alignment^53^. A phylogenic tree was generated from the filtered alignment using FastTree^56^. Because the relatively short read length of Illumina-generated sequences could reduce the resolution of taxonomic annotation against the GreenGenes reference database, the resulting OTU representative sequences were then searched against the Ribosomal Database Project naïve Bayesian classifier database (RDP 10 database, version 6)^58^, using the online program SEQMATCH (http://rdp.cme.msu.edu/seqmatch/seqmatch_intro.jsp). A confidence threshold of 0.80 was required. Singletons, defined here as taxa observed in only one sample, were removed as low-frequency reads offered no meaningful information in our experiment and instead added noise to the statistical models used. They were more likely to represent sequencing errors, contaminants, or transient organism without a biological role at the niche under study^57^. Singletons were discarded from the dataset using the ‘filter_otus_from_otu_table.py’ script in QIIME. Using OTU abundance and the corresponding taxonomic classifications, feature abundance matrices were calculated at different taxonomic levels, representing OTUs and taxa abundance per sample. The “Phyloseq”^59^, “Vegan”^60^ and “microbiome” R packages were used for the detailed downstream analysis on abundance matrix.

In the end, a total of 4,426,099 paired-end 250 bp reads were obtained, 3,611,320 of which were retained as high-quality sequences (Supplementary Table S10). On average, a total of 59,256 sequences per sample were achieved in the study, with a mean length of 441 ± 15 bp. These sequences were clustered into 8,229 non-singleton OTUs using the reference-based OTU-picking process. Among them, 7,185 were classified taxonomically down to the genus level (Supplementary Table S11). OTU counts per sample and OTU taxonomical assignments are available in Supplementary Table S11.

Relative abundance normalization was applied, which divides raw counts from a particular sample by the total number of reads in each sample.

#### Real-time quantitative PCR (qPCR) analysis of bacterial, anaerobic fungal and protozoan loads

Concentrations of protozoa, anaerobic fungi and bacteria in fecal samples were quantified using a QuantStudio 12 K Flex real-time instrument (Thermo Fisher Scientific, Waltham, USA). Primers for real-time amplification of protozoa (FOR: 5′-GCTTTCGWTGGTAGTGTATT-3′; REV: 5′-CTTGCCCTCYAATCGTWCT-3′), anaerobic fungi (FOR: 5′-TCCTACCCTTTGTGAATTTG-3′; REV: 5′-CTGCGTTCTTCATCGTTGCG-3′) and bacteria (5′-CAGCMGCCGCGGTAANWC-3′; REV: 5′-CCGTCAATTCMTTTRAGTTT-3′). Details on thermal cycles and the creation of standard curve for absolute quantification are reported in Mach *et al*.^17^ and Clark *et al*.^40^. To generate quantification curves, purified DNA was quantified using the Qubit dsDNA HS assay kit (Thermo Fisher Scientific®; Waltham, MA USA). This DNA was subsequently diluted serially by copy number and amplified using the 16S rRNA, the 18S rRNA or ITS1 qRT-PCR assays. The standard curve was included in each run. After each run, melting curve analysis was performed to the presence of the desired amplicon and to confirm the lack of primer dimers. In all cases, the melting curves analysis did not reveal any contamination due to genomic DNA or to non-specific amplification. Gel electrophoresis analysis of the PCR products also showed a single band of the expected size. The qPCR efficiencies covering the three amplicons were calculated in each run. For the ITS1 amplicon, efficiency values ranged from 1.902 to 1.988 with R^2^ value (square regression coefficient) > 0.95. In the assays targeting the 16S rRNA gene, efficiency values ranged from 1.861 to 1.918 with R^2^ value > 0.95, whereas in the assays targeting the 18S rRNA gene, efficiency values ranged from 1.888 to 1.959 with R^2^ value > 0.95.

### Nutrient intake estimation

The detailed dietary records for one month prior to fecal collection were performed through an in-depth interview with the horse’s owner or rider (similar to those reported in human microbiota studies)^61,62^. Briefly, for each animal, the proportion and type of hay, cereals, and commercial feed supplements were recorded and carefully reviewed by the research staff. The mean intakes of macro- and micronutrient intake from forage (mainly alfalfa-hay) and the different type of cereals consumed were then calculated based on the INRA food composition database for horses^63^. Additionally, the quantity of macro and micronutrient listed in the commercial feed supplements consumed according to the detailed descriptions of the respondents were obtained. The cereals (mainly based on barley and soybean meal) and the commercial feed supplements were considered as concentrates.

Then, the nutrient intake estimates of fat, protein, fiber, ash, as well as the MADC (g/d)^64^ and the total amount of UFC^64^ were estimated from the questionnaire by multiplying the frequency of the ingredient consumption by weight of an estimated average portion and nutrient content of the ingredient in question (Supplementary Table S12).

### Statistical analysis

#### Alpha diversity indices of the fecal microbiota

The α-diversity indexes were calculated using the “Phyloseq” and “microbiome” R packages from OTU abundance and relative genera abundance tables. The “microbiome” R package allowed us to study other global indicators of the gut ecosystem state, including measures of diversity, evenness, dominance, rarity, divergences and abundance. All samples were normalized using the *rarefy_even_depth* function in the “Phyloseq” R package, which is implemented as an *ad hoc* means to normalize microbiome counts that have resulted from libraries of widely differing size. During the alpha diversity step, singletons were kept in the analysis. Subsequently, singletons were removed.

#### Beta diversity of fecal microbiota

To estimate β-diversity, un-weighted and weighted UniFrac distances, as well as Bray-Curtis dissimilarity, were calculated from the OTU and the genera relative abundance tables. The β-diversity was visualized using PCoA, correspondence analysis (CA) and the NMDS with the “Phyloseq” and “ggplot2” R packages.

#### Description of the core microbiota

The core microbiome of individual samples was calculated using a detection threshold of 0.001% and a prevalence threshold of 99.9% (*e*.*g*. a given genera must be present in 99.9% of individuals with a relative abundance of at least 0.001%) in the “microbiome” R package.

#### Clustering of feces metagenomic samples into community types

The inter-individual variations in the gut microbiota composition was studied based on the conceptual framework of enterotypes, or more generically, community types^65^. According to this framework, the samples were clustered into bins based on their taxonomic similarity^66^. Briefly, clustering was performed with PAM^67^ using weighted UniFrac distance of the normalized OTUs counts. The optimal number of clusters or communities was chosen by the maximum average silhouette width, known as the silhouette coefficient (SC)^68^. The quality of those clusters or community types was assessed by the same measure, following the accepted interpretation that SC values above 0.5 indicate a reasonable clustering structure^69^. The graphical representation was performed through PCoA, CA and the non-negative matrix factorization (NMF) with the Kullback-Leibler dissimilarity^70^. To reinforce the robustness of the communities, we implemented the PAM with un-weighted UniFrac distance, Euclidean distance and Bray-Curtis dissimilarity on the normalized genera counts.

#### The contribution of each genus to the community type structure

The PLS-DA was used to identify the key genera responsible for the differences in the gut community types using “mixOmics” R package, coupled with the smallest *p*-values obtained from the Mann Whitney *U* test or the Fisher exact test. It is important to note that these *p-*values are only indicative, since *p*-values were computed on the same data used to define the two clusters. Furthermore, we explored the community type composition structure through two additional approaches: (i) the sparse-PLS-DA in a 10-fold cross-validation framework to identify the minimum significant discriminant genera that led to the same microbiota community clustering. The performance of PLS-DA, in selecting relevant predictors, was then being investigated by means of the area under the curve (AUC) of a receiver operating characteristic (ROC) curve. A test with a ROC-AUC of 1.0 is perfectly accurate, because the sensitivity is 1.0 (meaning that all relevant predictors were correctly identified, without irrelevant predictors wrongly assigned to the positive class); and (ii) the sparse *k*-means with the standardized Euclidean distance to identify the small fraction of genera that led to almost identical community clusters. In contrast to sparse PLS-DA, sparse *k*-means algorithm did not make use of predetermined clusters.

To explore whether the microbiota community structure was supported by a large number of genera, a cluster analysis based on Bray-Curtis distance was performed after removing the *k* highest rank genus defined by the PLS-DA loadings values. The genera with the highest-ranking cluster-discriminatory value among individuals were removed, starting from *k* = 1 until *k* = 100. For each *k*, the number of misclassified samples from the defined gut microbiota community types and the silhouette index were computed.

#### Network inference at the genus level for each community type

Networks at the genus level were inferred between community types. In order to prevent the compositional effects bias typical of the classical correlations methods, we calculated the correlations among genera using the PCIT approach, which identifies significant co-occurrence patterns through a data-driven methodology^71^. The genera with <0.1% mean relative abundances were excluded to acquire the results for the taxa that met the statistical conditions for correlation estimations. Nodes in the network represent the genera and edges that connect these nodes represent correlations between genera. Based on correlation coefficient and *p-*values for correlation, we constructed co-occurrence networks. The cutoff of *p-*values was 0.05. The cutoff of correlation coefficients was determined as r ≥ |0.35|. Network properties were calculated with the NetworkAnalyzer plugin in Cytoscape. We used the Cytoscape to visualize the network. Strong and significant correlation between nodes (r ≥ |0.60|) were represented with larger edge width in the network.

#### Association between gut microbiota, host, gut and environmental variables

The association between microbiota, host, gut and environmental variables was tested using both the microbiota community types and the relative genera abundance table.

First, we tested the hypothesis-driven associations between the gut community types and host, gut parameters and environmental variables using two different statistical approaches, namely: (i) the PERMANOVA test (a non-parametric method of multivariate analysis of variance based on pairwise distances) implemented in the *adonis2* function from “Vegan” R package. PERMANOVA allows testing the global association between community types and a group of variables, and (ii) the nonparametric Mann-Whitney *U* test for continuous variables and the Fisher exact test for categorical variables followed by BH multiple test correction. An adjusted *p*-value < 0.05 was considered as significant.

The host variables tested included: age, breed, sex, telomere length, host kinship, as well as endurance performance parameters such as biochemical and metabolome parameters at basal time and post-race, distance race covered, pulse rate on arrival at the veterinary check, the average speed during the race, the athletic performance ranking and whether the horse completed the race or was eliminated. The athletic performance ranking was categorized into the top 25% ranked performers, the individuals that arrived between the top 25% and 75% positions, the individuals that arrived in the last 25% positions and those eliminated from the race. The relative athletic performance ranking was computed among all ranked animals for each distance race (90, 120 and 160 km). To eliminate the inter-individual variability, the Δ values of biochemical and metabolic profiles (T1–T0) were considered.

The gut variables analyzed were the feces pH, the SCFA proportions in feces as well as the anaerobic fungal, bacterial and protozoan loads in feces, whilst the environmental variables included daily nutrient intakes corrected by UFC as well as hours traveled to arrive at the competition site, trainers, the stable from where they were coming, and the breeding establishment where they born and were kept together with their mothers until they were weaned. Additionally, we used two-way ANOVA to evaluate the relative effects of microbiota community types and race distance on the total observed variability in host, gut and environmental variables.

Second, covariates of microbiome variation (based on the relative genera abundance table) were identified by calculating the association between continuous host and gut phenotypes and genus-level community ordination (NMDS based on Bray-Curtis dissimilarity) with *envfit* function^72^ in the “vegan*”* R package, with 10,000 permutations^73^ and BH multiple testing correction. An adjusted *p*-value < 0.05 was considered as significant. This method enabled the selection of combined covariates with strongest correlation to microbiota variation.

#### Power analysis and effect size

A *post hoc* power analysis was conducted for the association between host and performance phenotypes and the gut microbiota community types. The power analysis for sex, breed, and risk of being elimination during the race was tested via Fisher exact test, whereas the Mann-Whitney *U* test was applied for age and athletic performance ranking. Fist, the power analysis was computed for an increasing sample size, while preserving the observed proportion of each gut community type among the cohort. Second, for the Fisher exact test with two classes (*e*.*g*. risk of being eliminated from the race) and for the Mann-Whitney *U* test applied to variables such as age and athletic performance ranking, the statistical power was computed for an increasing effect size, while preserving the observed sample size. For Mann-Whitney *U* test, we considered the skewed uniform distribution. The effect size considered was the ratio of the distribution mean over the two community types for Mann-Whitney *U*-test and the proportion of success over the two community types for Fisher exact test.

## Supplementary information

Supplementary information

Table S1

Table S2

Table S3

Table S4

Table S5

Table S6

Table S7

Table S8

Table S9

Table S10

Table S11

Table S12

## Data Availability

The metabolomic data is available at the NIH Common Fund’s Data Repository and Coordinating Center (supported by NIH grant, U01-DK097430) website, http://www.metabolomicsworkbench.org), where it has been assigned a Metabolomics Workbench Project ID: (UrqK1489). The data is directly accessible at: http://dev.metabolomicsworkbench.org:22222/data/DRCCMetadata.php?Mode=Study&StudyID=ST000945. The targeted locus study project has been deposited at DDBJ/EMBL/GenBank under the accession KBTQ00000000. The version described in this paper is the first version, KBTQ01000000. The bioproject described in this paper belongs to the BioProject PRJNA438436. The corresponding BioSamples accession numbers were SAMN08715709 to SAMN08715760.
