# Publisher Correction: Unraveling the effects of the gut microbiota composition and function on horse endurance physiology

**DOI:** 10.1038/s41598-020-69895-y

**Published:** 2020-09-23

**Authors:** Sandra Plancade, Allison Clark, Catherine Philippe, Jean-Christophe Helbling, Marie-Pierre Moisan, Diane Esquerré, Laurence Le Moyec, Céline Robert, Eric Barrey, Núria Mach

**Affiliations:** 1 MaIAGE, INRA, Université Paris-Saclay, Jouy-en-Josas, France; 2 ISBA, Université Catholique de Louvain, Louvain-la-Neuve, Belgium; 3 Gastroenterology Department, Vall d’Hebron Institut de Reserca, Barcelona, Spain; 4 UMR 1319, INRA, AgroParisTech, Université Paris-Saclay, Jouy-en-Josas, France; 5 UMR 1286, INRA, Université Bordeaux, Nutrition et neurobiologie intégrée, Bordeaux, France; 6 UMR 444, INRA, Plateforme GET, Castanet-Tolosan, France; 7 Unité de Biologie Intégrative et Adaptation à l’Exercice, UBIAE, EA7362, Université d’EvryUniversité Paris-Saclay, Evry, France; 8 UMR 1313, INRA, AgroParisTech, Université Paris-Saclay, Jouy-en-Josas, France; 9 Ecole Nationale Vétérinaire d’Alfort, Maisons-Alfort, France

Correction to: *Scientific Reports* https://doi.org/10.1038/s41598-019-46118-7, published on 03 July 2019

In the original version of this Article, the author Laurence Le Moyec was incorrectly indexed. This error has now been corrected.

