# Supplementary material for: Unraveling the effects of the gut microbiota composition and function on horse endurance physiology
Source: Sci Rep. 2019 Jul 3;9:9620. doi: 10.1038/s41598-019-46118-7 (PMC6610142; doi:10.1038/s41598-019-46118-7)

Unraveling the effects of the gut microbiota composition and function on horse endurance physiology

Sandra Plancade[1,2], Allison Clark[3], Catherine Philippe[4], Jean-Christophe Helbling[5], Marie-Pierre Moisan[5], Diane Esquerré[6], Laurence le Moyec[7], Céline Robert[8,9], Eric Barrey[8], and Núria Mach[8*]

Supplementary data

Population description

Sixty-one percent of the horses registered in the study were Arabian (n=32), whereas 31% (n=16) were Arabian cross horses and only 8% (n=4) of the horses were not Arabian. Breeds listed as Arabian cross in our cohort included Anglo-Arabian (AA), barbe Arabi (AB), riding horse, half Arabian (CS), Half Arabian, half unknown (NOC), cross breeding Anglo Arabian (AACR), and Arabian blood (DSA). For the 32 Arabian horses entered in the study, the mean ± SD age was 8.82 ± 1.78. There were 12 mares, 15 geldings and 5 stallions. A total of 12 (37%) out of 32 horses raced over 90 km, whereas 10 individuals (31%) raced over 120 km and 10 (31%) individuals raced over 160 km. Of the 32 horses, 6 (18%) were eliminated from the ride and 25% were classed within the top 10 performers. Similarly, the 16 Arabian cross horses were predominantly represented by mares and geldings with an average age of 9.30 ± 2.36 years. The Arabian cross individuals were also equally distributed to the three different race distances (from 31% to 37%). However, the rate of elimination (31%), and the individuals ranked within the top 10 performers (18%) were different compared to Arabian horses.

Orthogonal projections to latent structures (OPLS) analysis on metabolome profile

The OPLS analysis, which integrates an orthogonal signal correction (OSC), was applied to identify and characterize the relative abundance of metabolites identified from the Proton nuclear magnetic resonance (^{1}H NMR) that changed during the endurance exercise in the blood. The OSC-correction approach was conducted using DeviumWeb R package (https://github.com/dgrapov/DeviumWeb). Further details are explained elsewhere[4].

The 10-fold within model cross validation and permutation Monte Carlo testing (*n* = 1,000) were applied as another means of internally validating the OPLS model, *e.g.* whether the

model built with the samples were significantly better than any other models obtained by randomly permuting the original sample attributes. The quality of the O-PLS model was assessed by calculating the R^2 fit parameter and the Q^2 cross-validated coefficient of determination parameter. R^2 corresponds to the variance explained by the Y matrix. Q^2 (computed using the "leave-one-out" cross-validation method) estimates the model's predictability. $R^2 = 1$ indicates that the model describes the data perfectly, whereas $Q^2 = 1$ indicates perfect predictability. A loading plot was computed to illustrate the results of the OPLS model. In the loading plot, signals with a positive direction corresponded to metabolites that were present at high concentrations at post-ride samples relative to basal samples. The negative direction indicated metabolites with lower concentrations at post-ride compared to basal samples.

At basal time, the relative abundance of blood metabolites identified from the ^{1}H NMR analysis was within the normal range in all horse athletes (supplementary Table S8). Among the 51 metabolic peaks identified (including several amino acids, energy metabolism-related metabolites, saccharides, and organic osmolytes in the plasma), we observed two unassigned compounds at 4.0495-4.0395 and 4.4245-4.4095 ppm, respectively (supplementary Table S8). The post-race samples contained higher concentrations of lactate, acetate, 3-hydroxybutyrate, citrate, methyl functions of fatty acids, choline-containing components, as well as aromatic and branched amino acids (supplementary Fig. S12). Our OPLS model also showed that the horses' glycaemia was lower in post-race plasma samples than in pre-race samples, together with the concentrations of amino acids such as alanine and phenylalanine (supplementary Fig. S12). The explained variance, predictive capability and out-of-bag error of OPLS model remained higher ($R^2 = 0.64 \pm 3.54$; $Q^2 = 0.84 \pm 0.02$ and root mean square error of prediction (RMSEP) = 0.21 ± 0.09) than those of the 1,000-permutated models ($R^2 = 0.53 \pm 5.80$;

$Q^2 = 0.15 \pm 0.13$ and RMSEP = 0.56 ± 0.05) to discriminate pre- and post-endurance competition samples ($p < 0.001$).

Biochemical data

The overall basal biochemical profiles varied within the normal ranges for equine athletes[4,51] (supplementary Table S7). Post-race samples presented an above-normal total bilirubin, creatine kinase, aspartate transaminase, serum amyloid A concentrations, as well as non-esterified fatty acids and β-hydroxy-butyrate concentrations, reflecting hemolysis, muscular membrane permeability or inflammation and energy mobilization (supplementary Table S7).

Statistical power calculations and effect size for endurance performance

We performed the power computation to test the association between microbiota gut community types and the risk of being eliminated during the ride and athletic ranking performance in the competition. We analyzed: (i) the sample size that would allow reporting statistical significance for the observed effect size; and (ii) the effect size that would be statistically significant for the sample size of our cohort.

The proportions of eliminations among community type 1 and community type 2 were $\hat{p}_1 = 0.33\ (5/15)$ and $\hat{p}_2 = 0.16\ (6/36)$. The sample size that would allow reporting statistical significance for the observed effect size is depicted in supplementary Figure S9A. It displays the power of Fisher exact test at level of 0.05 for various sample size *n*, for the empirical proportions $\hat{p}_1$ and $\hat{p}_2$, and for the empirical proportion of each community type:

- $\mathrm{X}_1^{(k)} = \mathrm{Binom}(k \times n_1\, , \hat{p}_1)$
- $\mathrm{X}_2^{(k)} = \mathrm{Binom}(k \times n_2\, , \hat{p}_2)$
- $p_{fish}\left(X_1^{(k)}, X_2^{(k)}\right)$: Fisher exact test $\mathrm{p-value}$

- $p_k = \mathbb{P}\left[p_{fish}\left(X_1^{(k)}, X_2^{(k)}\right) \leq 0.05\right]$: power

A sample size of 330 individuals should be considered to reveal a significant association between microbiota communities and elimination with a power of 90%, given the empirical effect size.

The effect size that would be statistically significant for the sample size of our cohort is depicted in supplementary Figure S9C. The figure S9C displays the power of Fisher exact test at the level of 0.05 for the observed sample sizes, and for various proportions of eliminations. Power computation is implemented while preserving the overall proportion $\hat{p}_0$ of eliminations among the n = 51 animals:

$$p_0 = \frac{\hat{p}_1 n_1 + \hat{p}_2 n_2}{n}$$

and varying the proportions $\hat{p}_1$ and $\hat{p}_2$ of eliminations among each community type. Thus, the sample size of our cohort would have allowed us to detect an association between elimination and community type with a power of 0.9 for an effect size $\hat{p}_1/\hat{p}_2$ larger than 6, while the empirical effect size on our cohort is $\hat{p}_1\hat{p}_2$= 1.82.

We then performed power computation to test the association between community types and the athletic performance ranking in the competition. A total of $n_1' = 10$ animals and $n_2' = 30$ animals were ranked in community type 1 and type 2, respectively. In order to perform power computation of the Mann-Whitney test, one has to parameterize the distribution of the relative ranking. We considered a skewed uniform distribution D (μ) of mean μ on [0, 1], whose density is defined as:

$$f(x) = a_\mu x + b_\mu, \;\; a_\mu = 12\mu - 6, \;\; b_\mu = 4 - 6\mu$$

Smaller μ corresponds to smaller relative ranks, *e.g.* better performances. The empirical estimates of μ for community type 1 and type 2 were $\hat{\mu}_1 = 0.45$ and $\hat{\mu}_2 = 0.36$. Supplementary Fig. S9B displays the power of the Mann-Whitney test at the level of 0.05 for various sample sizes n, and for the empirical means and for the empirical proportion of each community type. A sample size of 700 should be considered to be able to find significant associations between athletic performance ranking and gut microbiota communities with a power 0.9, given the empirical effect size.

The supplementary Figure S9D displays the power of Mann-Whitney test at the level of 0.05 for the observed sample sizes, and for various values of μ. More precisely, power computation is implemented while preserving the value of μ over all ranked individuals as follows:

$$\hat{\mu}_0 = \frac{n_1\hat{\mu}_1 + n_2\hat{\mu}_2}{n_1 + n_2}$$

and varying the value of $\hat{\mu}_1$ and $\hat{\mu}_2$. Thus, the size of our cohort would allow to detect an association with power 0.9 for an effect size $\hat{\mu}_1/\hat{\mu}_2$ larger than 2.05, while the empirical effect size on our cohort is $\hat{\mu}_1/\hat{\mu}_2 = 1.22$.

Supplementary tables

Table S1. Metadata of horses recruited in the experiment.

The measured host properties (*e.g.* age, sex, breed) and athletic performance parameters (*e.g.* pulse rate, average speed during the race, distance covered, ranking and cause of elimination if any are described. Within breed: AA (Anglo-Arabian), AB (barbe Arabic), CS (riding horse, half Arabian), NOC (Half Arabian, half unknown), TF (French trotter), AACR (cross breeding Anglo Arabian), AR (Arabian), DSA (50% Arabian blood), and SHA (Shagya). Environmental factors such as the hours traveled to arrive at the competition site, the stable from where they were coming, the trainer and the breeding establishment where they were

born and kept together with their mothers until they were weaned are also described.

Table S2. Study of global indicators of the gut ecosystem state, including measures of evenness, diversity, dominance, rarity, divergences and abundance using "microbiome" R package.

Table S3. Relative abundance of each genus in our cohort.

Table S4. Differences in relative abundance of each genus between the two community types based on Fisher exact test and Mann-Whitney *U* test followed by Benjamini-Hochberg multiple test correction.

Table S5. Topological properties of the bacterial co-occurring network obtained from the gut community type 1 and community type 2, separately.

Table S6. Differences in short chain fatty acids (SCFA) proportions, bacterial, anaerobic fungal and protozoan loads in feces as well as feces pH and estimated diet nutrient intake between gut community types.

Table S7. Biochemical parameters obtained from 52 blood samples collected before and after the endurance race.

Table S8. Relative abundance of blood metabolites obtained from 52 individuals before and after the endurance race.

Table S9. Statistical effects of microbiota gut community types and distance race on the blood basal metabolites through a two-way ANOVA model.

Table S10. Summary of the study samples and fecal bacterial 16S rRNA gene amplicon sequence datasets.

Table S11. The OTU taxonomical assignments and OTU counts in each individual of the cohort.

Table S12. Estimated daily concentrate and forage intake, as well as daily macronutrient consumption for each horse recruited in the experiment.

The detailed dietary records for one month prior to fecal collection were obtained. For each animal, the proportion and type of hay, cereals, concentrate and commercial feed supplements were recorded. Then, the nutrient composition of each component in the diet was calculated based on INRA database to obtain the total amount of crude protein intake, crude fat intake, crude fiber intake, total ash intake, as well as the total amount of horse net energy value of feeds (UFC) and horse digestible crude protein (MADC; g/day) intake per day.

Supplementary figures

Figure S1. Experimental design and sampling.

A set of 52 endurance horses competing in one of the three-distance endurance competitions (90 km, 120 km and 160 km) were recruited with the owner's agreement. Before the endurance race (no more than 24 h before starting the race, in all cases), fecal samples were collected from all individuals to carry out pH, short chain fatty acid proportions, gut microbiota profiling and the quantification of anaerobic fungi, bacteria and protozoa loads. Blood samples were taken at basal time and immediately after the race to analyze biochemical and metabolomic profiles, as well as telomere length. The detailed dietary records for one month prior to fecal collection were obtained.

The picture of the horse was download from https://smart.servier.com/wp-content/uploads/2016/10/Animals.ppt. No changes were made. The image of the blood tube was download from https://smart.servier.com/smart_image/tube-20/. No changes were made. The analysis flask for feces collection was download from https://smart.servier.com/smart_image/culture-flask-4/ . No changes were made. The notepad image for dietary records was download from https://smart.servier.com/smart_image/notepad/. No changes were made.

Servier Medical Art by Servier is licensed under a Creative Commons Attribution 3.0 Unported License.

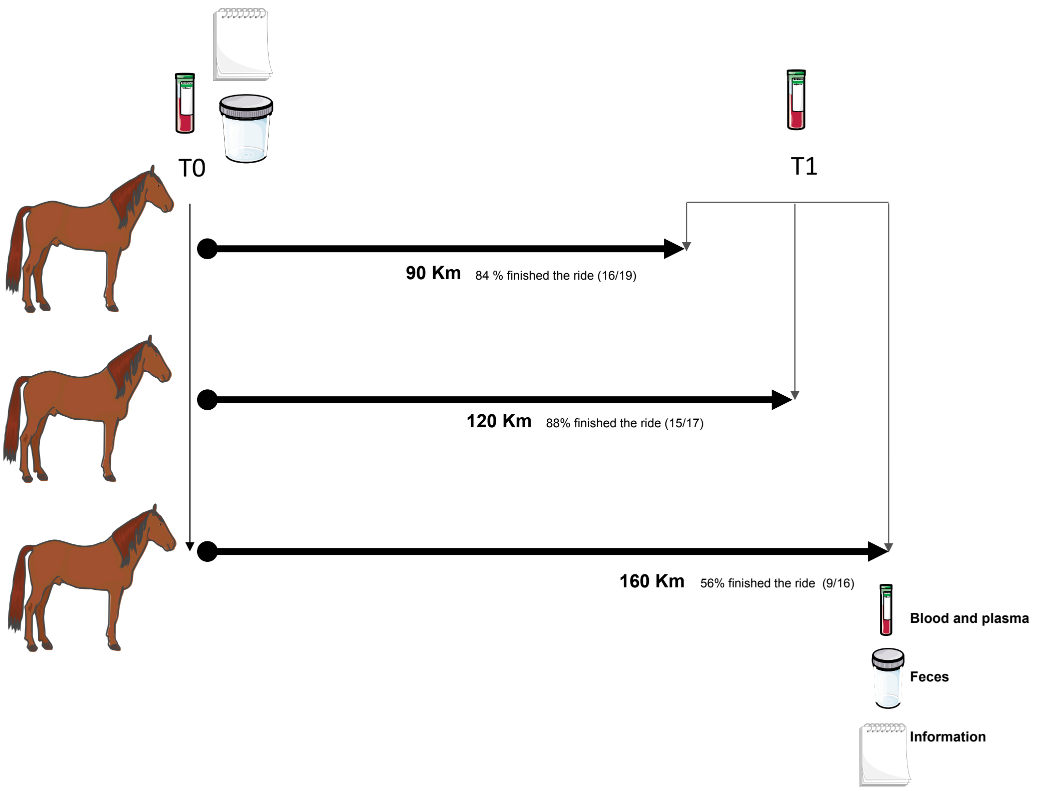
T0
T1
90 Km
84 % finished the ride (16/19)
120 Km
88% finished the ride (15/17)
160 Km
56% finished the ride (9/16)
Blood and plasma
Feces
Information

Figure S2. The endurance horse core microbiota.

(A) Box plot of the 23 genera shared by 99% of individuals in our cohort with a minimum detection threshold of 0.001%; (B) Heatmap of the endurance horse core microbiome. The X-axis shows the detection threshold of the core microbiota genera in our cohort regardless of the community type.

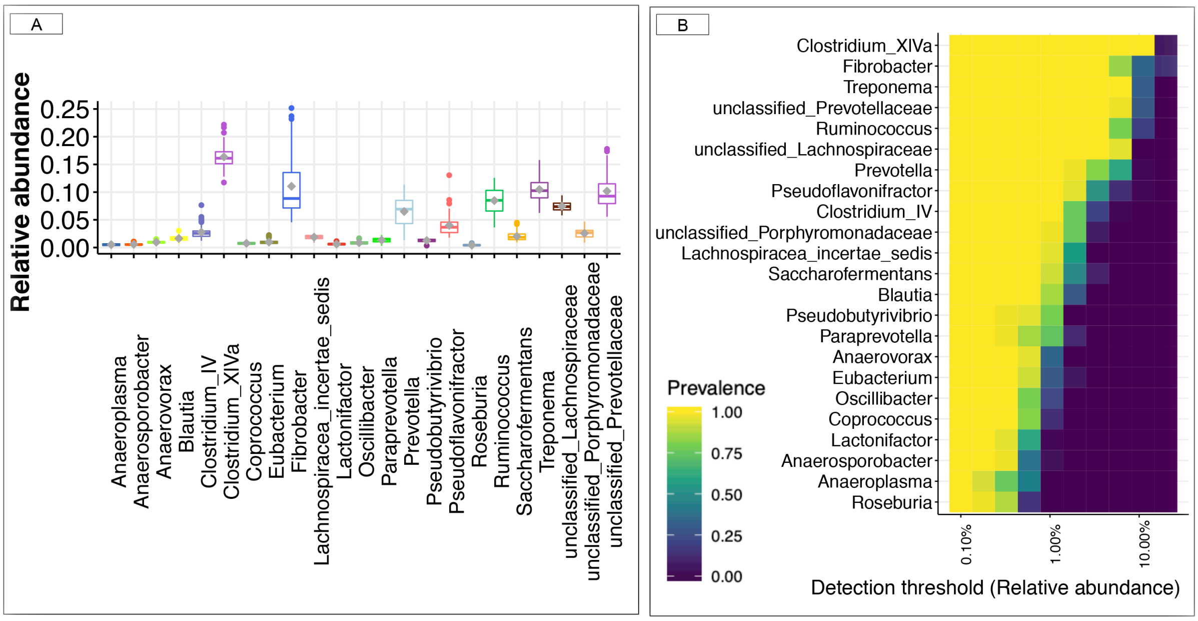

Figure S3. Estimation of β-diversity indexes from OTUs table in the 51 endurance horses. (A) Principal coordinate analysis (PCoA) with Bray-Curtis dissimilarity based on the OTUs abundance table; (B) Correspondence analyses (CA) with weighted UniFrac distance based on the OTUs abundance table. Both CA axes 1 and 2 were plotted. Together they explained 29.9% of whole inertia; (C) PCoA with Bray-Curtis dissimilarity based on the relative genera abundance table; (D) Non-negative matrix factorization (NMF) decomposition with the Kullback-Leibler dissimilarity based on the relative genera abundance table. In all cases, color indicates the community type: community type 1 (green) and community type 2 (purple).

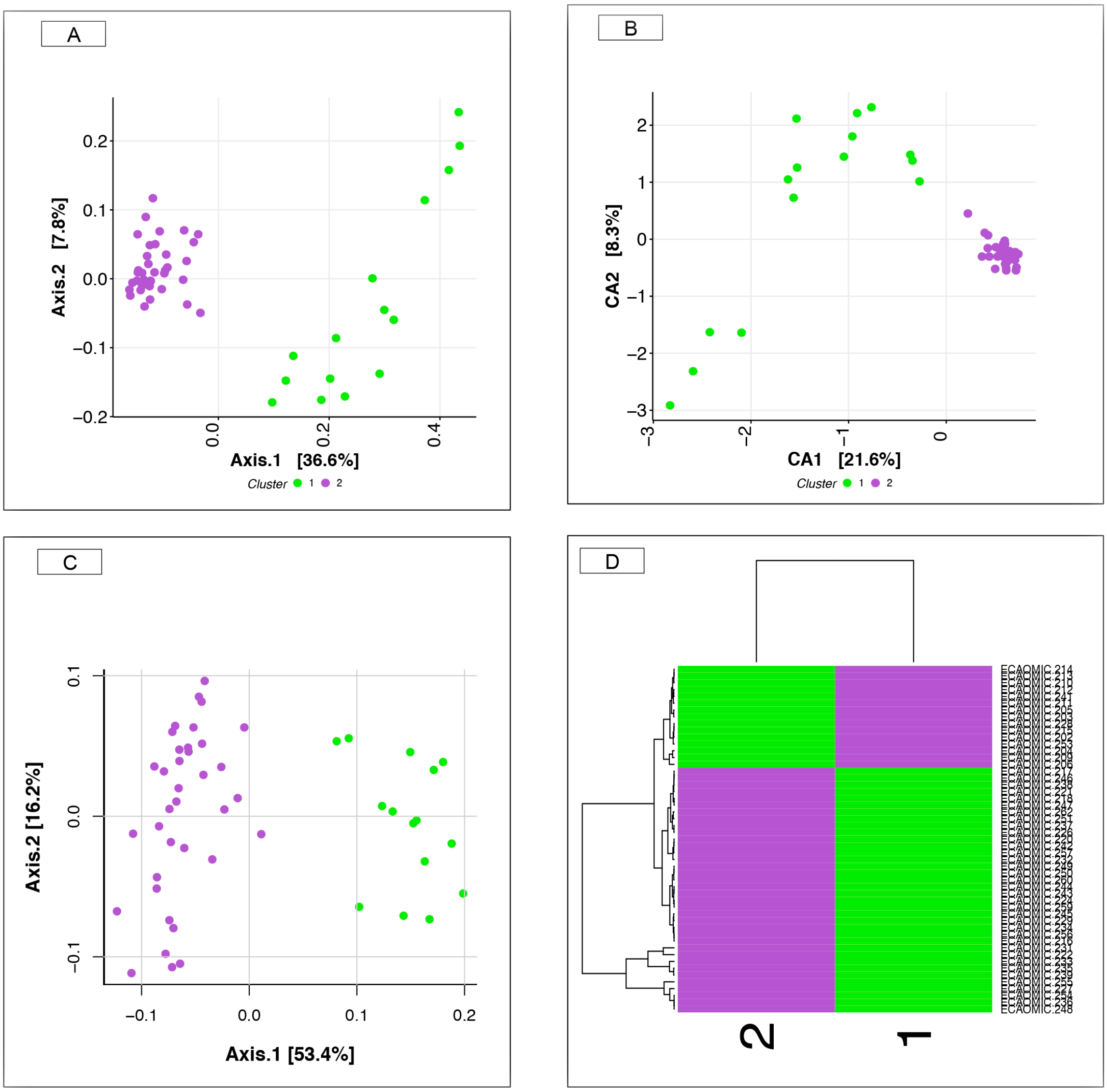

Figure S4. Host and performance parameters for each gut microbiota community type. (A) Bar plot of breeds represented in each of the two communities' types. For each microbiota community type, the bar plot represents the number of individuals ascribed to each breed: AA (Anglo-Arabian; red), AB (barbe Arabic; green), CS (riding horse, half Arabian; orange), NOC (Half Arabian, half unknown; brown), TF (French trotter; grey), AACR (cross breeding Anglo Arabian; blue), AR (Arabian; purple), DSA (50% Arabian blood; yellow), and SHA (Shagya; pink); (B) Bar plot of sex represented in each of the two communities' type. For each microbiota community type, the bar plot represents the number of individuals ascribed to each sex: female (green); gelding (purple) and male (blue); (C) Bar plot of distance covered in each of the two communities' types. For each microbiota community type, the bar plot represents the number of individuals ascribed to each race distance covered: 90 km (green); 120 km (blue) and 160 km (orange); (D) Bar plot of age ranges represented in each of the two communities' types. For each microbiota community type, the bar plot represents the number of individuals ascribed to each age ranges: > 6 and < 9 years old (green); > 9 and < 12 years old (purple) and > 15 years old (blue); (E) Lollipop plot of the individual average speed for the entirety of the race for each community type. The bars were ordered following their average speed values. In all cases, color indicates the community type: community type 1 (green) and community type 2 (purple); (F) Lollipop plot of the individual pulse rate on arrival at the veterinary check. The bars were ordered following their average pulse values. In all cases, color indicates the community type: community type 1 (green) and community type 2 (purple); (G) Bar plot of the endurance race ranking and the animals being eliminated during the competition for each community type. For each microbiota community type, the bar plot represents the number of individuals ascribed to each endurance race ranking: top 25% ranked performers (pink), the individuals that arrived between the top 25% and 75%

positions (yellow), the individuals that arrived in the last 25% positions (turquoise), and those eliminated from the race (green).

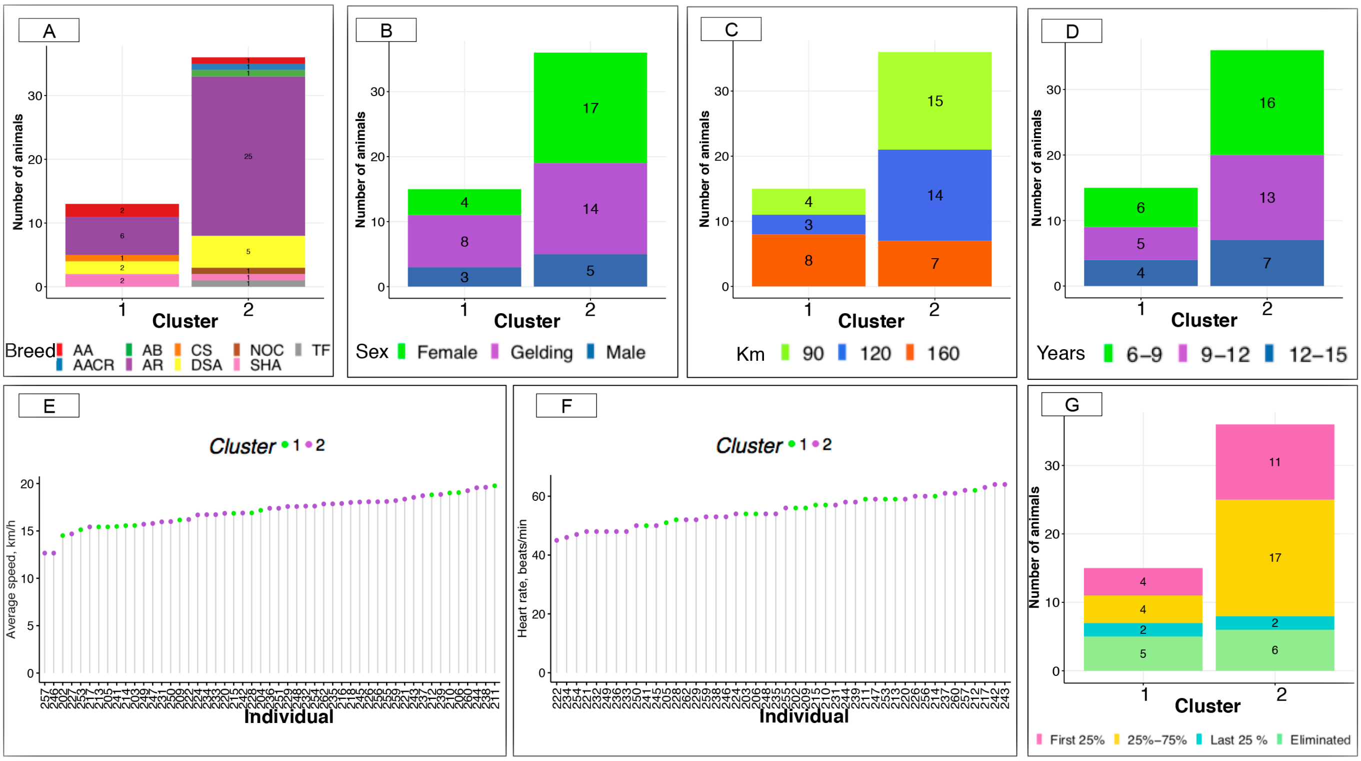

Figure S5. Measures of diversity, evenness, dominance, rarity, divergences and abundance between gut community types.

All measures plotted were significantly different between community types (adjusted *p*-value < 0.05, Mann-Whitney *U* test followed by Benjamini-Hochberg multiple test correction). In all cases, boxes show median and interquartile range, and whiskers indicate 5th to 95th percentile. The box color indicates the community type: community type 1 (green) and community type 2 (purple).

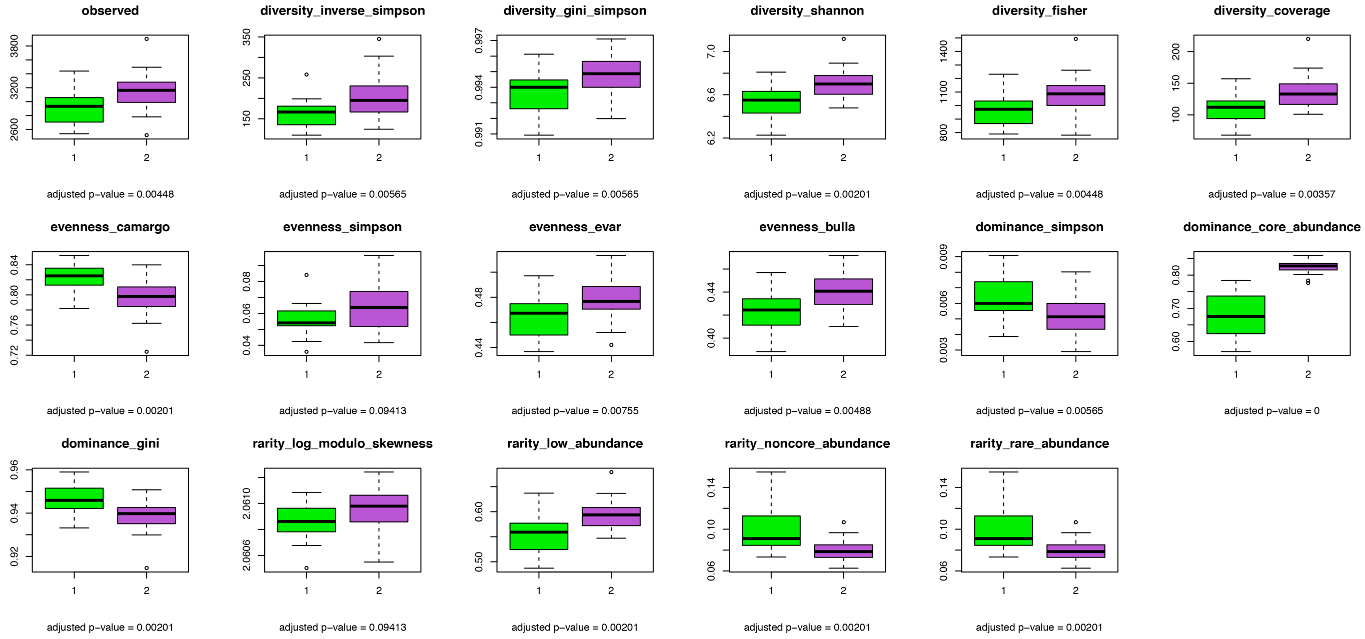

Figure S6. Robustness of the gut microbiota community type clustering.

(A) Relationship between the *p*-values obtained by combining the Mann-Whitney *U* test and Fisher exact test, and the partial least squares discriminant analysis (PLS-DA) model loadings values for each genus; (B) The performance of cross-validation PLS-DA models in selecting relevant predictors was investigated by means of the area under the curve (AUC). Only those genera that were relevant to the response, meaning that they had nonzero loading values, were considered; (C) Number of misclassified samples in the sparse *k*-means clustering for several values of the number of non-zero loadings; (D) Principal coordinate analysis (PCoA) with Bray-Curtis dissimilarity based on relative genera abundances after removal of the *k* genera with the highest PLS-DA loadings. A *k* of 10, 20, 30,and 50 was considered. In all cases, color indicates the community type: community type 1 (green) and community type 2 (purple).

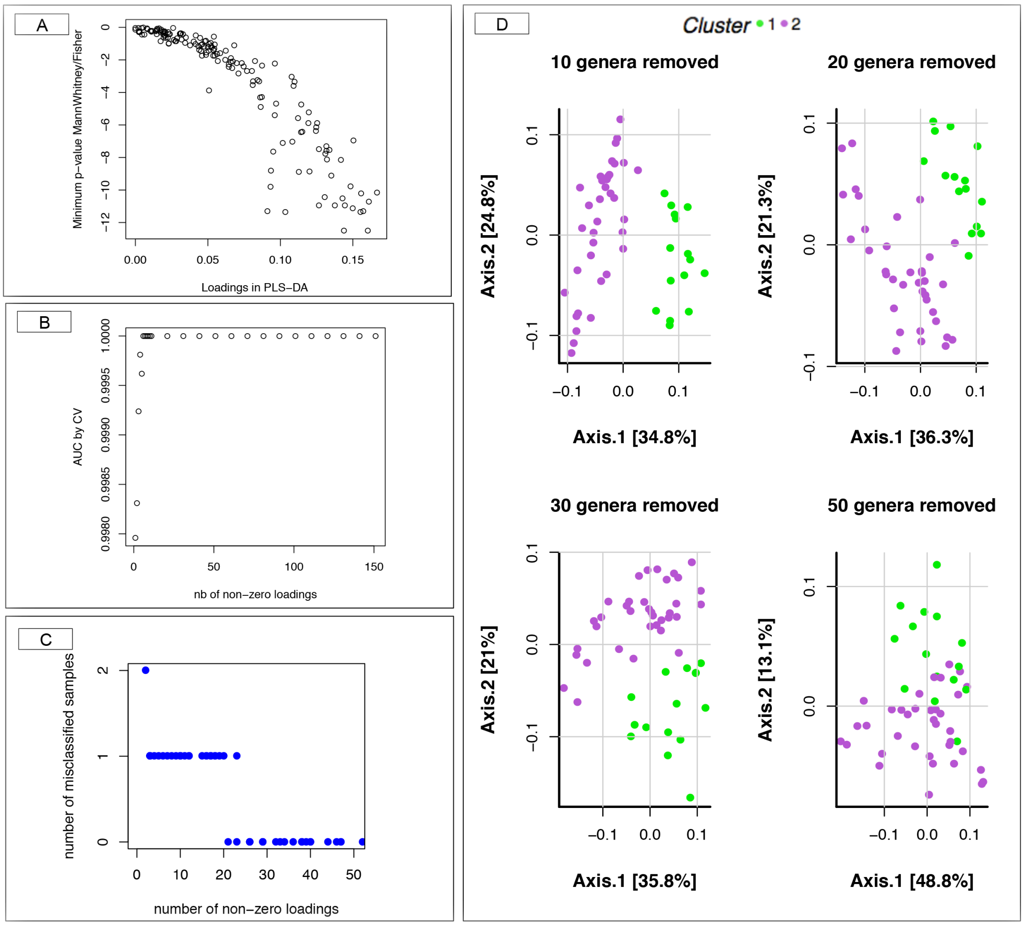

Figure S7. Differential metabolite expression profiles in horse plasma.

^{1}H NMR spectra of horse plasma samples collected before the race (green color) and afterwards (purple). The **Δ** spectra is colored in blue. The main metabolites are labeled as follows 1: methyl moieties from fatty acids, 2: branched amino acids (valine, leucine, isoleucine), 3: methylene moieties from fatty acids, 4: lactate, 5: alanine, 6: acetate, 7: glutamate and glutamine, 8: citrate, 9: creatine, 10: choline containing compounds, 11: glucose, 2: aromatic amino acids (tyrosine and phenylalanine).

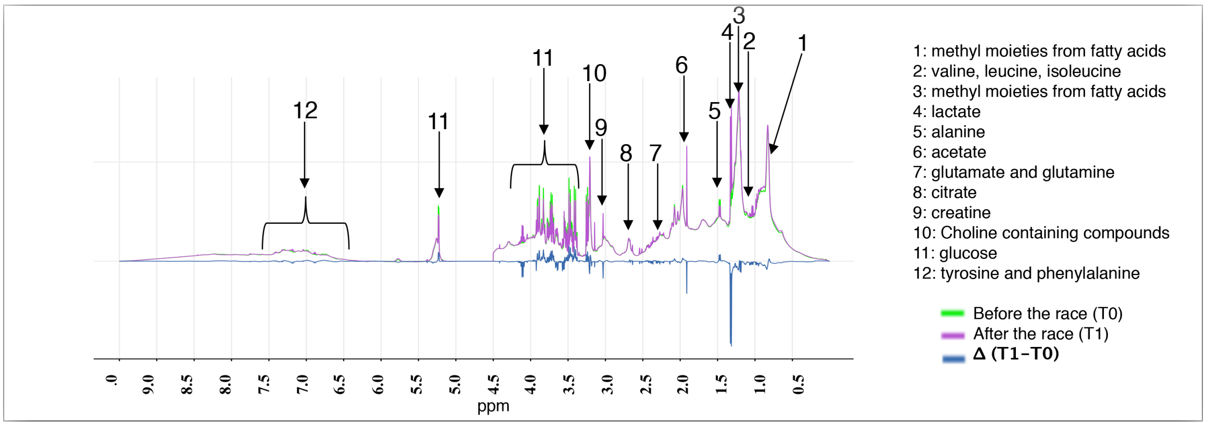

Figure S8. Relationship between the gut microbiota and the endurance performance parameters

(A) Distribution of eliminated and non-eliminated animals using the Principal coordinate analysis (PCoA) with weighted-UniFrac distance. Dot color indicates the individuals eliminated during the competition (green) and non-eliminated during the competition (purple); (B) The mosaic plot represents the link between the microbiota community types and the endurance race ranking. The relative frequencies of individuals from community type 1 and community type 2 for each ranking category are represented: top 25% ranked performers (pink), the individuals that arrived between the top 25% and 75% positions (yellow), the individuals that were classified as the last 25% ranked performers (turquoise), and those eliminated from the race (green).

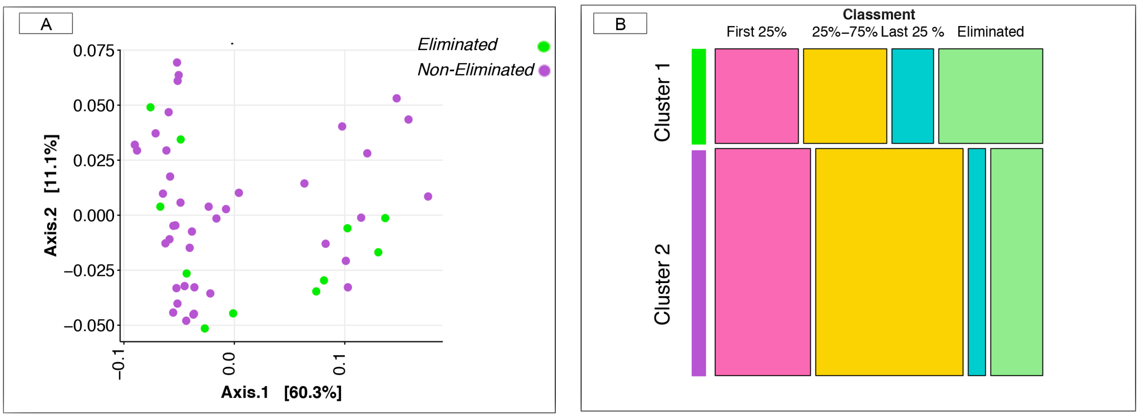

Figure S9. Statistical power calculation and effect size for the risk of being eliminated during the race and athletic ranking performance

The power computation was used to tests the association between gut microbiota community types and two animal performance criteria: risk of being eliminated during the race and athletic ranking performance. (A-B) Sample size that would allow reporting statistical significance for the observed effect size; (C-D) Effect size that would be statistically significant for the sample size of our cohort. The probability to detect an association between the risk of being eliminated during the competition or the athletic ranking performance and the gut community types at level 0.05 was calculated as a function of the cohort size for the observed effect size and proportion of each community. The Fisher exact test and Mann-Whitney *U* test were used respectively.

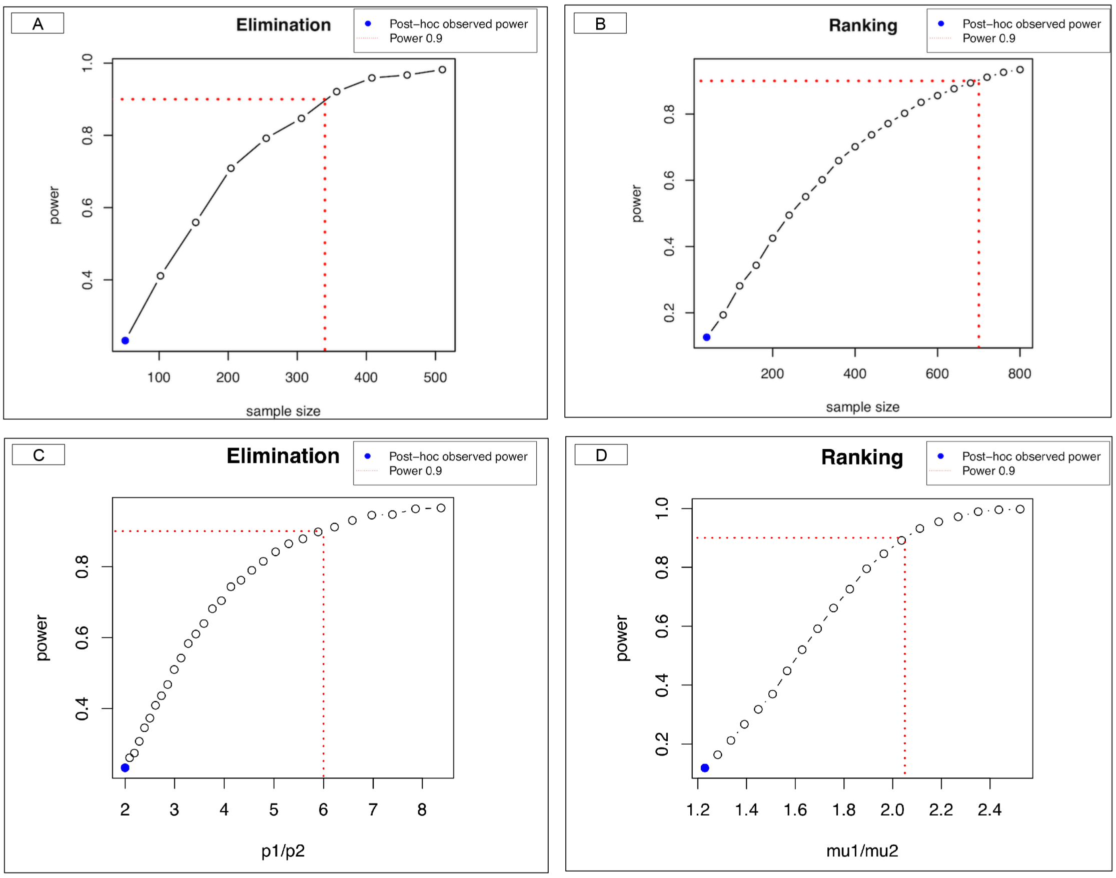

Figure S10. Pedigree plot of the 52 endurance horses in the experiment.

A six-generation pedigree plot is illustrated, with different shapes for male (squares) and female (circles). The shapes are black for the 52 horses in the study.

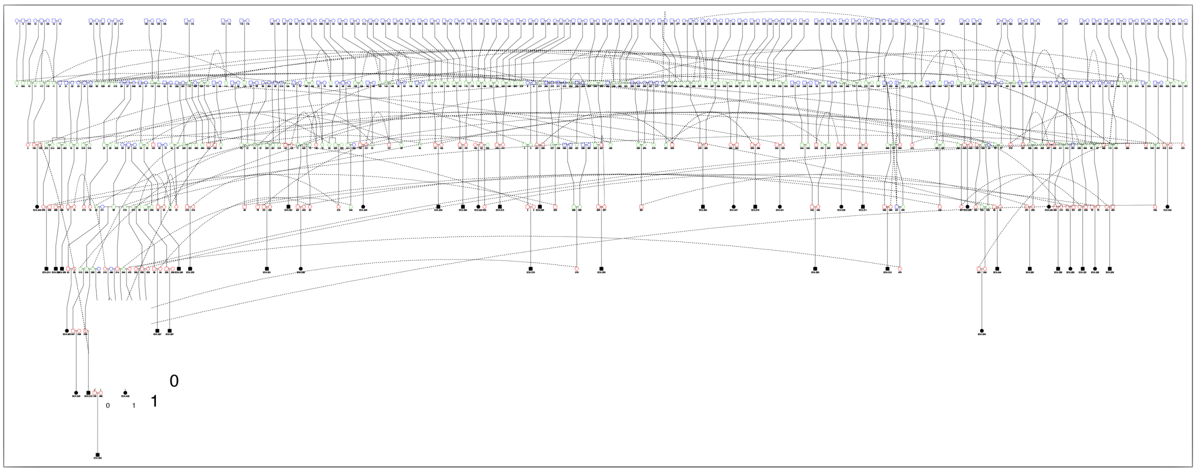

Figure S11. Genetic kinship matrix between the 52 endurance horses in the experiment. Heatmap of the kinship coefficient matrix. Each entry in the matrix is the kinship coefficient between two subjects. Animals are arranged in the order of their genetic relatedness. Genetically similar animals are near each other. Note that the diagonal elements did not have values above unity, showing no consanguineous mating in the families. The community type (1 or 2) is delineated below to the animal name. In the heatmap, red = high values of genetic relatedness, white = low values of genetic relatedness.

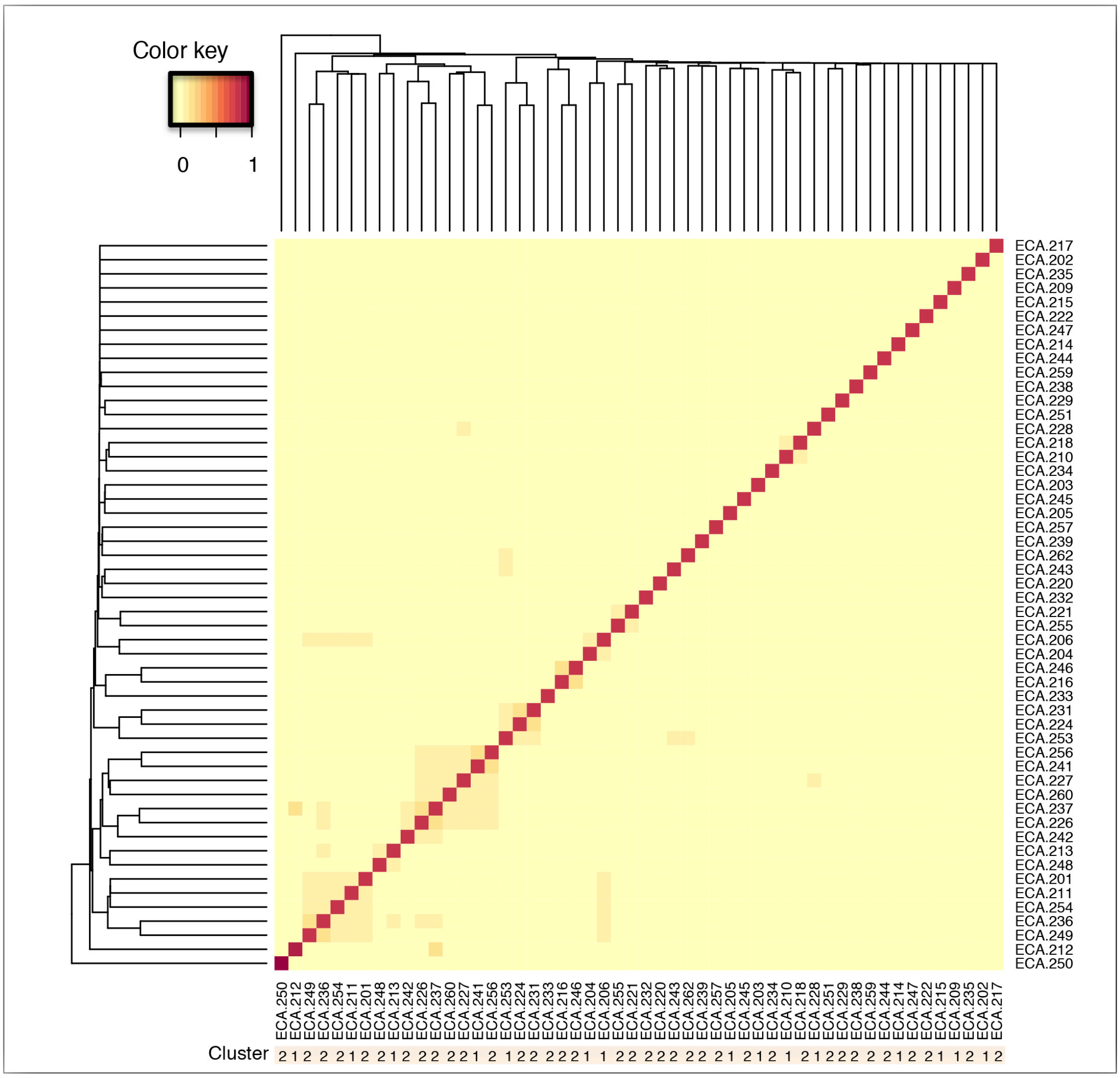

Figure S12. Differential metabolite expression profiles in horse plasma using OPLS.

(A) The orthogonal projections to latent structures (OPLS) loading plot, which represents the enhanced metabolites in plasma in pre- and post- endurance competition samples. A positive loading score indicates there was a relatively greater levels of the metabolite in post-endurance competition samples and a negative loading score indicates a relatively lower levels of the metabolite, with respect to pre- endurance competition samples. The model was calculated considering 1 orthogonal signal corrected (OSC) latent variables partial least squares discriminant analysis (PLS-DA) model.

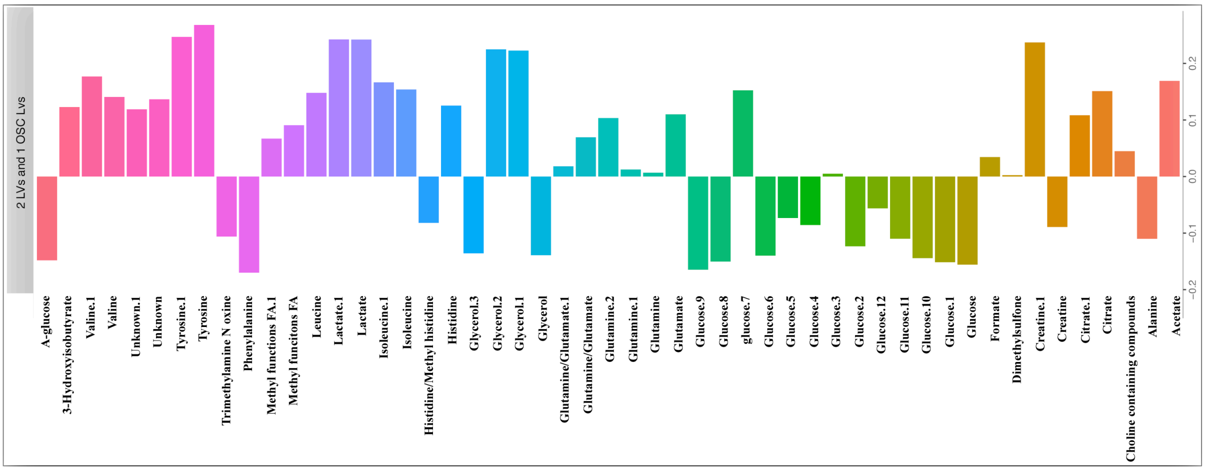

Supplement: Supplementary file 1 — Supplementary information [file 41598_2019_46118_MOESM1_ESM.pdf]
